# Regulation of Inducible Potassium Transporter KdpFABC by the KdpD/KdpE Two-Component System in *Mycobacterium smegmatis*

**DOI:** 10.3389/fmicb.2017.00570

**Published:** 2017-04-24

**Authors:** Maria K. Ali, Xinfeng Li, Qing Tang, Xiaoyu Liu, Fang Chen, Jinfeng Xiao, Muhammad Ali, Shan-Ho Chou, Jin He

**Affiliations:** ^1^State Key Laboratory of Agricultural Microbiology, College of Life Science and Technology, Huazhong Agricultural UniversityWuhan, China; ^2^Biotechnology Program, Department of Environmental Sciences, COMSATS Institute of Information TechnologyAbbottabad, Pakistan; ^3^Institute of Biochemistry and NCHU Agricultural Biotechnology Center, National Chung Hsing UniversityTaichung, Taiwan

**Keywords:** *Mycobacterium smegmatis*, two-component system (TCS), KdpD/KdpE, KdpFABC, Kdp-ATPase, potassium transporter, K^+^ limitation

## Abstract

Kdp-ATPase is an inducible high affinity potassium uptake system that is widely distributed in bacteria, and is generally regulated by the KdpD/KdpE two-component system (TCS). In this study, conducted on *Mycobacterium smegmatis*, the *kdpFABC* (encoding Kdp-ATPase) expression was found to be affected by low concentration of K^+^, high concentrations of Na^+^, and/or ${\text{NH}}_{4}^{+}$ of the medium. The KdpE was found to be a transcriptional regulator that bound to a specific 22-bp sequence in the promoter region of *kdpFABC* operon to positively regulate *kdpFABC* expression. The KdpE binding motif was highly conserved in the promoters of *kdpFABC* among the mycobacterial species. 5′-RACE data indicated a transcriptional start site (TSS) of the *kdpFABC* operon within the coding sequence of *MSMEG_5391*, which comprised a 120-bp long 5′-UTR and an open reading frame of the 87-bp *kdpF* gene. The *kdpE* deletion resulted in altered growth rate under normal and low K^+^ conditions. Furthermore, under K^+^ limiting conditions, a single transcript (*kdpFABCDE*) spanning *kdpFABC* and *kdpDE* operons was observed. This study provided the first insight into the regulation of *kdpFABC* operon by the KdpD/KdpE TCS in *M. smegmatis*.

## Introduction

Potassium ion (K^+^) is a ubiquitous monovalent cation that is essential for all living cells. It plays important roles in a wide variety of cellular functions such as turgor pressure maintenance, osmo-regulation, acid-base homeostasis, membrane potential adjustment, enzyme activation, and gene expression regulation (Booth, 1985; Epstein, 1986, 2003; Dinnbier et al., 1988; Stumpe et al., 1996; Follmann et al., 2009). Generally, a higher intracellular concentration of K^+^ (150–500 mM) is required than that of extracellular environment (Epstein and Schultz, 1965; Dinnbier et al., 1988) to efficiently carry out these functions. Hence, cell has to precisely regulate the uptake of K^+^ for its growth and survival in various hostile conditions (Stumpe et al., 1996; Kuo et al., 2005). For this purpose, animal cells are usually equipped with Na^+^/K^+^ ATP pumps that are generally missing in bacterial cells (Heitzmann and Warth, 2008; Gumz et al., 2015), whereas several K^+^ channels and active transporters are present in the prokaryotic cells to maintain potassium homeostasis. These bacterial K^+^ uptake systems vary a lot in their structures and affinities for the cation, which ultimately help bacteria to precisely maintain intracellular K^+^ concentrations under diverse environmental conditions (Altendorf et al., 1992, 1998; Epstein, 2003; Nanatani et al., 2015). In prokaryotes, three major families of K^+^ uptake transporters, Trk/Ktr/HKT, Kup/HAK/KT, and Kdp, have been discovered (Buurman et al., 1995; Ballal et al., 2002, 2007; Berry et al., 2003; Sato et al., 2014). Besides these, some K^+^ channels have also been reported (Epstein, 2003). Among these K^+^ uptake transporters, Trk and Kup are constitutively expressed in *Escherichia coli*, with the uptake rate of Trk system nearly 10 times higher than that of Kup transporter (Rhoads et al., 1976). On the contrary, the Kdp-ATPase is an inducible system, exhibiting high affinity for K^+^ uptake that is specific to bacterial and archeal kingdoms (Diskowski et al., 2015). This system becomes operational under low K^+^ concentrations (~2 μM as estimated from the *K*_*m*_-value; Epstein, 2016) when other K^+^ uptake systems such as Trk and Kup fail to function (Epstein, 1992). Generally, Kdp-ATPase complex is consisted of four subunits, encoded in the *kdpFABC* operon. Among these subunits, KdpF is required for stabilizing the complex (Altendorf et al., 1998; Gassel et al., 1999), KdpA is involved in the binding and translocation of K^+^ (Altendorf et al., 1992; Greie, 2011), while KdpB is associated with KdpC that is essential for the ATP hydrolysis (Haupt et al., 2005; Greie and Altendorf, 2007). In most cases, *kdpFABC* is found adjacent to *kdpDE*, which encodes proteins that are involved in the regulation of Kdp-ATPase system. The inducible Kdp-ATPase is regulated by histidine kinase KdpD and response regulator KdpE, which constitute a typical two-component system (TCS) (Walderhaug et al., 1992), with KdpD responding to a multitude of signals such as low K^+^ concentrations, osmotic pressure upshift, different intracellular ATP levels, and pH of the medium (Asha and Gowrishankar, 1993; Jung and Altendorf, 2002; Hamann et al., 2008; Epstein, 2016). Previously, it was suggested that turgor pressure was another stimulus for *kdpDE* induction (Laimins et al., 1981), however recent observations did not support this idea (Epstein, 2016). Other than its role in K^+^ homeostasis, KdpD/KdpE TCS is also well-reported for its role in the regulation of bacterial virulence in diverse bacterial species including *Mycobacterium tuberculosis, Staphylococcus aureus, Salmonella enterica* serovar Typhimurium and *Pseudomonas aeruginosa* (Parish et al., 2003; Alegado et al., 2011; Xue et al., 2011; Feinbaum et al., 2012; Freeman et al., 2013). Moreover, as a regulator, KdpE was found to regulate many essential genes involved in key metabolisms, stress tolerance and virulence (Njoroge et al., 2013; Burda et al., 2014; Samir et al., 2016; Parker et al., 2017).

The Kdp-ATPase and its cognate TCS (KdpD/KdpE) have been well-characterized in *E. coli* (Walderhaug et al., 1992; Gassel et al., 1998; Brandon et al., 2000; Heermann and Jung, 2010; Laermann et al., 2013; Epstein, 2016). Cytoplasmic stimuli such as K^+^ concentration, ionic strength and ATP content are perceived by histidine kinase KdpD (Jung and Altendorf, 2002; Heermann and Jung, 2010). After detecting these stimuli, KdpD experiences auto-phosphorylation, with its phosphoryl group transferred to the transcriptional regulator KdpE (Voelkner et al., 1993). The phosphorylated KdpE (KdpE~P) binds to a 23-bp T-rich sequence in the promoter of *kdpFABC* with stronger affinity as compared to un-phosphorylated KdpE (Nakashima et al., 1992, 1993; Sugiura et al., 1992). Moreover, some accessory proteins such as UspC (Heermann et al., 2009) and enzyme IIA^Ntr^ (Lüettmann et al., 2009) have also been reported for their interactions with KdpD for signal transduction and cross talk. Among pathogenic bacteria, KdpD/KdpE TCS has been well-studied in *S. aureus* (Xue et al., 2011). The *kdpFABC* genes were significantly up-regulated (up to 100-fold) under salt stress in this osmo-tolerant bacterial species (Price-Whelan et al., 2013). In *S. aureus*, KdpE also regulates the expression of many other genes. Among these are the capsular polysaccharide genes (*cap*) that play a vital role in the pathogenicity of *S. aureus*. It was found that KdpE specifically bound to the *cap* promoter region and deletion of *kdpDE* resulted in the decreased transcription of *cap* genes (Zhao et al., 2010). Expression of other virulence factors such as *spa, hla, aur, geh*, and *hlgB* was also regulated by KdpE, and direct binding of KdpE to the promoter regions of many of these genes, has been observed (Xue et al., 2011).

Based on sequence homology to *E. coli*, Kdp-ATPase was found to be widely distributed among different bacterial and archeal genera (Ballal et al., 2007; Heermann and Jung, 2010). In case of genus *Mycobacterium*, the Kdp system has been found in *M. tuberculosis, M. avium, M. bovis, M. smegmatis, M. marinum*, and others, except *M. leprae* and *M. ulcerans* (Cholo et al., 2008; Bretl et al., 2011). Among these species, *M. smegmatis* is a fast growing and comparatively safe bacterial species to work with, which is now commonly used as a surrogate for *M. tuberculosis*. Generally, *M. smegmatis* is perceived as a non-pathogenic species. However, some investigations have demonstrated its pathogenic potential (Wallace et al., 1988; Alvarado-Esquivel et al., 2009; Best and Best, 2009; Driks et al., 2011; Xu et al., 2013). Up to now, KdpD/KdpE TCS in *M. smegmatis* has not been well-characterized. Due to the different organizations of *kdpFABC* and *kdpDE* in various mycobacterial species, investigation of this system in *M. smegmatis* would be valuable. As KdpD/KdpE is involved in regulating K^+^ homeostasis, elucidation of its detailed function and regulatory mechanism for its downstream target genes would provide better options to cope with mycobacterial infections. Moreover, due to the structural differences between the K^+^ uptake systems of prokaryotes and eukaryotes (Cholo et al., 2008), the current system may present a potential target for antibiotic development against mycobacterial infection. In the current study, the promoter of *kdpFABC* was clearly identified and the regulation of *kdpFABC* expression by KdpD/KdpE TCS under different potential stimuli was investigated in detail. To the best of our knowledge, for the first time this study provided strong evidences that KdpE bound to the promoter region of *kdpFABC* operon (P*kdpF*) at a specific site in *M. smegmatis*. In addition, we also predicted the coding sequence of KdpF in *M. smegmatis* as well as in many other mycobacterial species.

## Materials and methods

### Bacterial strains and growth conditions

Strains and plasmids used in this study are listed in Table 1. *E. coli* strains DH5α and BL21 (DE3) were grown in Lysogeny broth (LB) media. *M. smegmatis* wild type strain MC^2^155 and its mutants were grown at 37°C in Middlebrook 7H9 broth (Difco Becton Dickinson, USA) supplemented with 0.2% (v/v) glycerol and 0.05% (v/v) Tween80 or on Middlebrook 7H10 agar (Difco Becton Dickinson, USA) supplemented with 0.5% (v/v) glycerol (Tang et al., 2014). For potassium limitation study, Hartmans-de Bont (HdB) medium [35 μM EDTA, 490 μM MgCl_2_. 6H_2_O, 7 μM CaCl_2_, 0.8 μM NaMoO_4_. 2H_2_O, 5.49 μM MnCl_2_. 2H_2_O, 6.95 μM ZnSO_4_. 7H_2_O, 1.68 μM CoCl_2_. 6H_2_O, 20 μM FeSO_4_. 7H_2_O, 0.8 μM CuSO_4_. 5H_2_O, 7.08 mM NaH_2_PO_4_, 8.9 mM K_2_HPO_4_, or 8.9 mM Na_2_HPO_4_, 15 mM (NH_4_)_2_SO_4_, supplemented with 0.2% (v/v) glycerol and 0.05% (v/v) Tween 80, pH 7.0] (Smeulders et al., 1999; Hartmans et al., 2006) was used. Kanamycin (30 μg/mL), hygromycin B (100 μg/mL), 5-bromo-4-chloro-3-indolyl-β-D-galactopyranoside (X-gal) (50 μg/mL) and 10% sucrose (w/v) were added in media when required.

**Table 1:** **List of the plasmids and bacterial strains used in this study**.

**Plasmids and bacterial strains**	**Relevant characteristics**	**Purposes**	**References**
**STRAINS**			
DH5α	*E. coli*	Cloning host	Lab stock
BL21(DE3)	*E. coli*	Protein expression host	Lab stock
BL21(DE3)*-*pET28-*kdpE*	BL21(DE3) with pET28-*kdpE*	Over-expression of *kdpE*	This work
MC^2^155	A wild-type *M. smegmatis* strain		ATCC
MC^2^155/pMV261	*M. smegmatis* MC^2^155 with plasmid pMV261		This work
*ΔkdpD*	*kdpD* mutant strain of MC^2^155	Gene knockout	This work
*ΔkdpE*	*kdpE* mutant strain of MC^2^155	Gene knockout	This work
C*ΔkdpE*	*ΔkdpE* complementary with pMV261-*kdpE*	Gene complementary	This work
Ms/P*5391*::*lacZ*	MC^2^155 harboring pMV261-P*5391*::*lacZ*	β-galactosidase assay	This work
Ms/P*kdpD*::*lacZ*	MC^2^155 harboring pMV261-P*kdpD*::*lacZ*	β-galactosidase assay	This work
*ΔkdpE*/P*5391*::*lacZ*	*ΔkdpE* harboring pMV261-P*5391*::*lacZ*	β-galactosidase assay	This work
*ΔkdpE*/P*kdpD*::*lacZ*	*ΔkdpE* harboring pMV261-P*kdpD*::*lacZ*	β-galactosidase assay	This work
Ms/P*ttg*::*lacZ*	MC^2^155 harboring pMV261-P*ttg*::*lacZ*	β-galactosidase assay	This work
Ms/P*ttga*::*lacZ*	MC^2^155 harboring pMV261-P*ttga*::*lacZ*	β-galactosidase assay	This work
Ms/P*gtg*::*lacZ*	MC^2^155 harboring pMV261-P*gtg*::*lacZ*	β-galactosidase assay	This work
Ms/P*gtga*::*lacZ*	MC^2^155 harboring pMV261-P*gtga*::*lacZ*	β-galactosidase assay	This work
Ms/P*null*::*lacZ*	MC^2^155 harboring pMV261-P*null*::*lacZ*	β-galactosidase assay	Tang et al., 2014
Ms/P*hsp*::*lacZ*	MC^2^155 harboring pMV261-P*hsp*::*lacZ*	β-galactosidase assay	Tang et al., 2014
*ΔkdpD*/P*ttg*::*lacZ*	*ΔkdpD* harboring pMV261-P*ttg*::*lacZ*	β-galactosidase assay	This work
*ΔkdpD*/P*ttga*::*lacZ*	*ΔkdpD* harboring pMV261-P*ttga*::*lacZ*	β-galactosidase assay	This work
*ΔkdpD/*P*gtg*::*lacZ*	*ΔkdpD* harboring pMV261-P*gtg*::*lacZ*	β-galactosidase assay	This work
*ΔkdpD*/P*gtga*::*lacZ*	*ΔkdpD* harboring pMV261-P*gtga*::*lacZ*	β-galactosidase assay	This work
*ΔkdpD*/P*null*::*lacZ*	*ΔkdpD* harboring pMV261-P*null*::*lacZ*	β-galactosidase assay	This work
*ΔkdpD*/P*hsp*::*lacZ*	*ΔkdpD* harboring pMV261-P*hsp*::*lacZ*	β-galactosidase assay	This work
*ΔkdpE*/P*ttg*::*lacZ*	*ΔkdpE* harboring pMV261-P*ttg*::*lacZ*	β-galactosidase assay	This work
*ΔkdpE*/P*ttga*::*lacZ*	*ΔkdpE* harboring pMV261-P*ttga*::*lacZ*	β-galactosidase assay	This work
*ΔkdpE*/P*gtg*::*lacZ*	*ΔkdpE* harboring pMV261-P*gtg*::*lacZ*	β-galactosidase assay	This work
*ΔkdpE*/P*gtga*::*lacZ*	*ΔkdpE* harboring pMV261-P*gtga*::*lacZ*	β-galactosidase assay	This work
*ΔkdpE*/P*null*::*lacZ*	*ΔkdpE* harboring pMV261-P*null*::*lacZ*	β-galactosidase assay	This work
*ΔkdpE*/P*hsp*::*lacZ*	*ΔkdpE* harboring pMV261-P*hsp*::*lacZ*	β-galactosidase assay	This work
**PLASMIDS**			
pMD19-T simple vector	Amp^R^	Cloning vector	Takara (Japan)
pET28a(+)	Kan^R^, T7-driven	Expression vector	Novagen
pET28-*kdpE*	*kdpE* between BamHI/HindIII sites of pET28a(+)	Over-expression of *kdpE*	This work
pMV261	Kan^R^, pAL5000 replicon, colE1 replicon, *hsp60* promoter	Expression vector	Yang et al., 2012
pMV261-P*5391*::*lacZ*	The candidate promoter of *MSMEG_5391* (500-bp upstream of the *MSMEG_5391* start codon) with *lacZ* in XbaI and NheI sites of pMV261	β-galactosidase assay	This work
pMV261-P*kdpD*::*lacZ*	The candidate promoter of *kdpDE* (500-bp upstream of the *kdpD* start codon) with *lacZ* in XbaI and NheI sites of pMV261	β-galactosidase assay	This work
pMV261-P*ttg*::*lacZ*	XbaI-300-bp candidate promoter of *MSMEG_5391*-TTG-HindIII::*lacZ* of pMV261	β-galactosidase assay	This work
pMV261-P*ttga*::*lacZ*	XbaI-300-bp candidate promoter of *MSMEG_5391*-TTGA- HindIII::*lacZ* of pMV261	β-galactosidase assay	This work
pMV261-P*gtg*::*lacZ*	XbaI-300-bp candidate promoter of *kdpFABC*-TTGn_120_-GTG-HindIII::*lacZ* of pMV261	β-galactosidase assay	This work
pMV261-P*gtga*::*lacZ*	XbaI-300-bp candidate promoter of *kdpFABC*-TTGn_120_-GTGA -HindIII::*lacZ* of pMV261	β-galactosidase assay	This work
pMV261-P*null*::*lacZ*	*lacZ* in XbaI and NheI sites of pMV261	β-galactosidase assay	Tang et al., 2014
pMV261-P*hsp*::*lacZ*	*lacZ* in HindIII and NheI sites of pMV261, which has the promoter of *hsp60*	β-galactosidase assay	Tang et al., 2014
pMind	Kan^R^, Hyg^R^, pAL5000 replicon, colE1 replicon	Gene knockout	Blokpoel et al., 2005
pGoAL17	Kan^R^, pBR322 replicon	Gene knockout	Parish and Stoker, 2000
pMind-*kdpD* U′D′ SDV	Suicide delivery vector of *kdpD*	Gene knockout	This work
pMind-*kdpE* U′D′ SDV	Suicide delivery vector of *kdpE*	Gene knockout	This work

### Construction of Δ*kdpD* and Δ*kdpE* mutants and functional complementation of Δ*kdpE*

The mutant strains of *M. smegmatis* were constructed by the method of homologous recombination as described earlier (Yang et al., 2012). The detailed procedures used to construct *kdpD* and *kdpE* mutants (Δ*kdpD* and Δ*kdpE*) and the *kdpE* complementary strain (CΔ*kdpE*) are provided in Supplementary Material (Figures S1–S3).

### Purification of KdpE protein

The *kdpE* gene was amplified and cloned into pET28a(+) (Novagen) to construct expression plasmid pET28-*kdpE*. The 6-His-tagged KdpE protein was heterologously expressed in *E. coli* BL21(DE3)-pET28-*kdpE* (Table 1) and purified with Ni-NTA affinity column (GenScript). Purified KdpE protein was concentrated and desalted by MWCO 3000 Amicon ultra centrifugal filters (Millipore). The purity of KdpE was analyzed on 12% SDS-PAGE (Figure S4) and quantified by Bradford assay (Bradford, 1976).

### Electrophoretic mobility shift assay (EMSA)

DNA probes were amplified with and without 6-FAM-labeled primers (Table S1) and the probes were purified with a gel purification kit (Bioteke). Labeled probes were incubated with different amounts of KdpE in EMSA binding buffer [10 mM Tris (pH 7.5 at 20°C), 100 mM KCl, 1 mM EDTA, 0.1 mM DTT, 5% v/v glycerol, 0.010 mg/mL BSA] (Hellman and Fried, 2007) with the addition of 0.5 mM Na_2_ATP to activate phosphorylation of KdpE. Unlabeled probes were used for competition experiments. The reaction mixture was incubated at 25°C for 1 h and then subjected to 6% native polyacrylamide gel electrophoresis at 150 V for 1 h in a 0.25 × Tris-borate-EDTA (TBE) running buffer. The gel image was obtained using Typhoon Scanner (GE Healthcare).

### DNase-I foot printing

To determine the binding sequence of KdpE in the P*kdpF*, the fluorescence labeled probes and the reaction system were the same as used in EMSA. The mixtures were treated with 0.02 U DNase-I for 2 min. Further procedures of purification and analysis of samples were same as described previously (Tang et al., 2014).

### RNA extraction from *M. smegmatis* and synthesis of cDNA

To study gene expression, bacterial cells were grown in HdB media (with and without K^+^) to the late logarithmic phase. Total RNA was extracted by TRIzol method (Schmittgen and Livak, 2008) and the samples were analyzed on 1% agarose gel for qualitative assessment and NanoDrop 2000 (Thermo Scientific, USA) spectrophotometer was used for the quantitative analysis. First-strand cDNA was constructed with the commercially available PrimeScript RT reagent kit with gDNA Eraser (Takara Biotechnology, Japan) according to manufacturer's instructions.

### Real time qPCR

For real time qPCR reaction (RT-qPCR), gene specific primers were used (Table S1). Appropriate dilutions of cDNA were prepared and used in RT-qPCR. The reaction was performed in real-time PCR machine (Lightcycler 480 instrument II, Roche) with following conditions: 45 cycles of 95°C for 10 s, 63°C for 10 s, and 72°C for 10 s. Relative quantification of the gene expression was determined by 2^−ΔΔCT^ method (Livak and Schmittgen, 2001). The *sigA* (*MSMEG_2758*) was used as a reference gene for the determination of relative expression.

### 5′-rapid amplification of cDNA ends (5′-race)

To identify the transcriptional start site (TSS) of *kdpFABC* operon, 5′-RACE analysis was performed with RNA extracted from *M. smegmatis* cells grown in HdB media (with and without K^+^) using 5′-Full RACE kit (Takara biotechnology, Japan) according to manufacturer's instructions and as reported in our previous studies (Tang et al., 2014, 2016). The P*kdpF* region was amplified by reverse transcriptase (RT) PCR from RACE cDNA libraries using 5′-RACE outer primers and P*kdpF* specific outer primers (Table S1). The resulting amplified product was purified from the agarose gel and used as template in next amplification step with 5′-RACE inner primers along with P*kdpF* specific inner primers (Table S1). Finally, 250 bp sequence obtained was purified and ligated into pMD19-T (Takara biotechnology, Japan) and mobilized into *E. coli* DH5α. Clones were sequenced and the first nucleotide next to 5′-RACE adaptor was considered to be the TSS of the operon.

### β-galactosidase assay

All strains were grown in HdB (with and without K^+^) and 7H9 broth with different concentrations of salts (NaCl, NH_4_Cl and KCl), sucrose and with different pH-values containing kanamycin (30 μg/mL) at 37°C for 20 h (late-logarithmic phase). The collected culture was assayed for β-galactosidase activity as previously described (Bharati et al., 2013).

### Effects of different stress conditions on the growth of *M. smegmatis* strains

*M. smegmatis* MC^2^155 and its mutant derivatives were subjected to various stress conditions such as temperature, pH, and salt stress. Previously described methodology was used to introduce stress conditions to the bacterial strains (Gebhard et al., 2008). For each stress experiment, bacterial cells were grown to mid-logarithmic phase in the 7H9 medium supplemented with 0.2% glycerol and 0.05% Tween 80. The specific stimulation conditions are described in Table S2.

### Bioinformatics analysis

Promoter sequences of *kdpFABC* in various mycobacterial species were found by NCBI blastn (https://blast.ncbi.nlm.nih.gov/Blast.cgi) and KEGG server (http://www.genome.jp/kegg/). The multiple sequence alignments of KdpF proteins and KdpE binding motifs were conducted using BioEdit program, and final output was prepared by ESPript 3.0 server (http://espript.ibcp.fr/ESPript/ESPript/) (Robert and Gouet, 2014). DNA logo was generated using program WebLogo (http://weblogo.berkeley.edu/) (Crooks et al., 2004).

## Results

### Expression of *kdpFABC* operon under K^+^ limiting condition and its regulation by the KdpD/KdpE TCS

**Figure 1:**
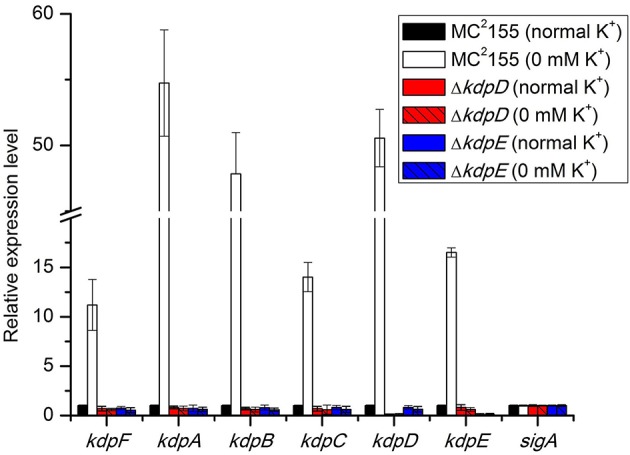
**Relative expression levels of ***kdp*** loci in response to growth in K^**+**^ limiting and non-limiting conditions**. Cultures of wild type, as well as Δ*kdpD* and Δ*kdpE* mutants were grown to late exponential phase in the presence and absence of K^+^ in HdB medium. Total RNA was extracted and cDNA was used for quantitative analysis. Data represent the averages of biological triplicates. Error bars indicate standard deviation. *sigA* (*MSMEG-2758*) gene was used as reference gene for the determination of relative expression levels of *kdpFABC* and *kdpDE* genes in K^+^ limiting condition with respect to normal K^+^ condition (wild type strain). Normal K^+^ stands for HdB medium where K^+^ concentration was similar to that defined earlier (Smeulders et al., 1999), which was ~17.8 mM as measured by flame photometry, whereas 0 mM K^+^ stands for HdB medium in which K^+^ salt was replaced by similar concentration of Na^+^ salt and the residual K^+^ concentration was ~23 μM as measured by flame photometry.

Initially, the relative expression levels of *kdpFABC* and *kdpDE* genes were studied in bacterial strains grown in the presence and absence of K^+^ in the medium. In wild type MC^2^155, the expression of above genes occurred at a very basal level under normal K^+^ condition whilst considerably higher levels were observed in 0 mM K^+^ condition (Figure 1). In both Δ*kdpD* and Δ*kdpE* mutants, no expression levels of *kdpFABC* genes were detected compared to the wild type MC^2^155 growing under normal K^+^ condition (Figure 1). This indicated that expression levels of *kdpFABC* were controlled by KdpD/KdpE TCS and were up-regulated under K^+^ limiting condition.

### Co-transcription of *kdpFABC* and *kdpDE* operons under low k^+^ condition

Next, we sought to investigate the transcription patterns of *kdpFABC* and *kdpDE* operons. RT-PCR analysis showed an obvious amplification band in the region spanning *kdpC* (last gene of the *kdpFABC* operon) and *kdpD* (first gene of the *kdpDE* operon) when cDNA from wild type strain grown under K^+^ limiting condition was used as a template (Figure S5). In contrast, no amplification band was detected with cDNA from wild type strain grown under normal K^+^ condition in HdB medium (data not shown). These results showed that in *M. smegmatis*, the *kdpFABC* and *kdpDE* operons were co-transcribed as a single transcript (*kdpFABCDE)* from P*kdpF* under K^+^ limiting condition but not under normal K^+^ condition.

### Binding of KdpE protein to P*kdpF*

**Figure 2:**
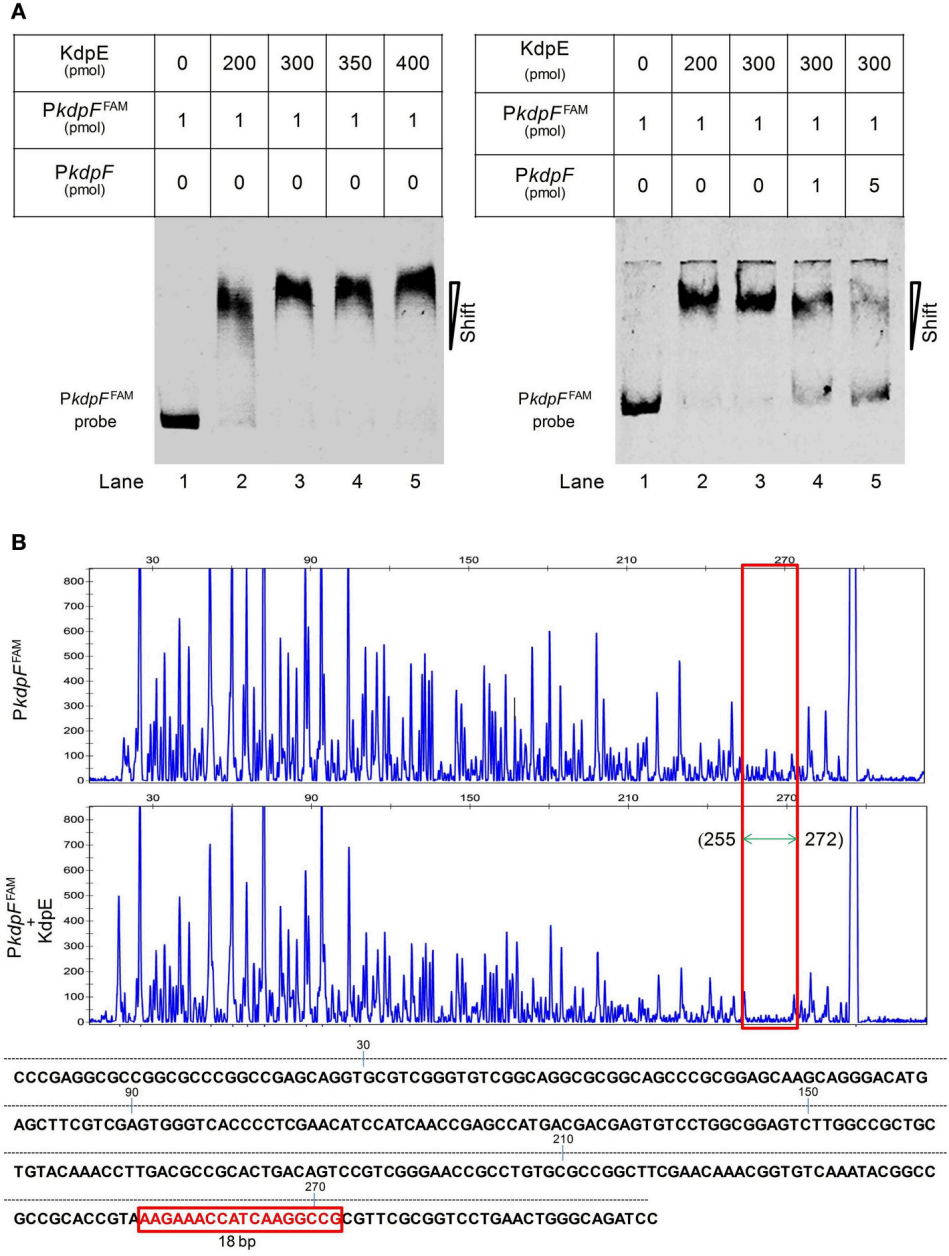
**Binding of purified KdpE protein to specific DNA motif in the P***kdpF***. (A)** EMSA of KdpE with P*kdpF*. Left lane 1: 6-FAM-labeled probe of P*kdpF*, left lanes 2–5: complex formation with increasing concentrations of KdpE. Right lane 1: 6-FAM-labeled probe of P*kdpF*, right lanes 2–3: complex formation with increasing concentrations of KdpE, right lanes 4–5: competition with increasing concentration of unlabeled probe. **(B)** Electropherograms indicating protected DNA region in P*kdpF* after DNase-I digestion of 6-FAM-labeled probe in the absence of KdpE (upper panel) and in the presence of KdpE (lower panel). The fluorescence signals of 6-FAM-labeled fragments are plotted against probe length. The protected region in electropherograms and the 18-bp DNA motif of KdpE are boxed in red. The 300-bp DNA probe used in the assay is shown below with numerical values along the DNA sequence representing the positions of nucleotides with reference to the above graphical scale.

To gain further insights into the regulation of *kdpFABC* operon, we determined the binding of KdpE to the promoter P*kdpF*. The *in vitro* EMSA of KdpE with the candidate promoter of *kdpF* was carried out in the presence of ATP, and a dramatic shift of the labeled DNA probe in the presence of KdpE was observed (Figure 2A). It was noteworthy that such binding was relatively weaker in the absence of ATP (data not shown), indicating phosphorylation of KdpE was required for efficient promoter binding. The EMSA was also carried out in a competition way to reveal the specific binding of KdpE with the P*kdpF*; when unlabeled P*kdpF* probe was added to the reaction mixture in increasing proportions, the unlabeled probe clearly competed with the labeled probe, causing labeled probe to move to the position of no protein binding (Figure 2A). In *S. aureus*, KdpE was reported to bind not only to the promoter region of *kdpFABC* but also to that of *kdpDE* (Xue et al., 2011). However, no such binding was observed when we performed the EMSA experiment with the promoter of *kdpDE* operon (P*kdpD*) (data not shown).

In order to further verify the role of KdpE as a transcriptional regulator, a β-galactosidase assay was performed using *lacZ* as the reporter gene. Surprisingly, no β-galactosidase activity was obtained when 500-bp upstream of *MSMEG_5391* (P*5391*) (*MSMEG_5391* which is present upstream of *kdpA* gene and it has been annotated to translate hypothetical protein) was selected as the candidate promoter (Figure S6A). On the other hand, very basal level activities were detected with the P*kdpD* without any significant difference between the wild type and Δ*kdpE* under normal and limiting K^+^ conditions (Figure S6B).

### Minimum KdpE binding motif in the P*kdpF*

**Figure 3:**
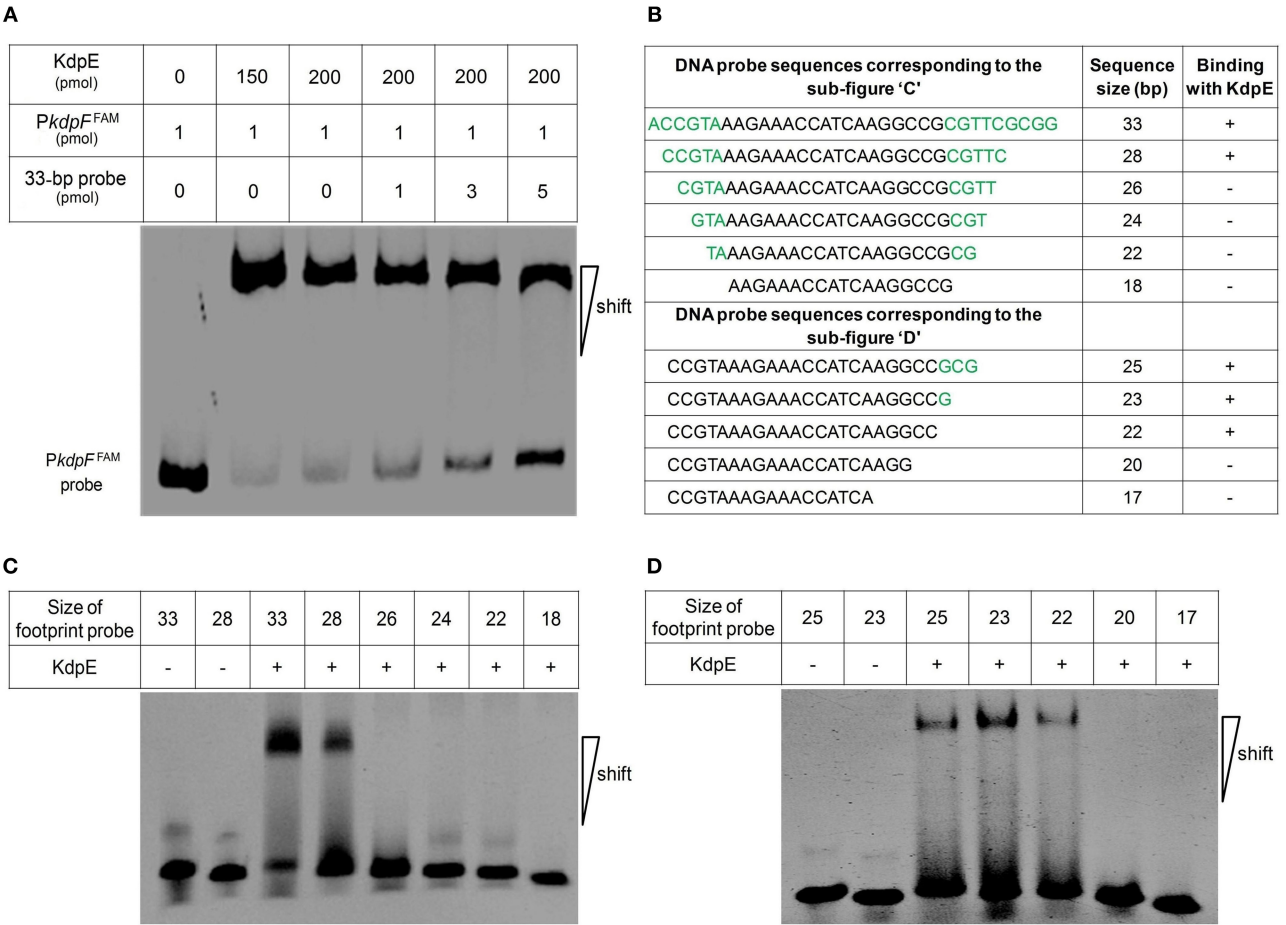
**Determination of minimum DNA sequence required for successful binding of KdpE protein with P***kdpF***. (A)** EMSA competition assay performed with labeled (300-bp 6-FAM-labeled probe) and unlabeled 33-bp DNA probes. With increasing concentration of unlabeled 33-bp probe, the band intensity of labeled probe with KdpE protein decreased while that of unlabeled probe increased, indicating that unlabeled 33-bp probes could effectively compete with the 300-bp 6-FAM-labeled probe in binding KdpE. **(B)** The sizes and sequences of different DNA probes used for the determination of exact foot print sequence. **(C)** Shorter double stranded DNA probes used (based on DNase-I foot printing results) in EMSA to determine the minimum DNA sequence required for KdpE binding with P*kdpF*. Sizes of DNA probes were decreased by removing few nucleotides on both ends of the 33-bp probe to determine minimum nucleotides required for binding. **(D)** Unnecessary nucleotides (based on DNA sequence homology with other mycobacterial species) were trimmed down from the 3′ end of the 28-bp probe to determine the minimum sequence required for KdpE-DNA interaction.

Above EMSA data provided clear evidences for the *in vitro* binding between KdpE and candidate P*kdpF*. To map the precise binding sequence of KdpE, a DNase-I foot printing assay was performed using the 300-bp 6-FAM-labeled promoter fragment of the *kdpFABC* operon. It is clear from the electropherograms that a short 18-bp sequence (5′-AAGAAACCATCAAGGCCG-3′) appeared to be protected from DNase-I digestion as a result of KdpE binding (Figure 2B). To validate this result, the EMSA experiment was further performed using essentially the same 18-bp sequence (mentioned above). Unexpectedly, we failed to obtain binding between the 18-bp probe identified from DNase-I foot printing and KdpE protein (data not shown). We thus extended the 18-bp probe with few base pairs flanking the central 18-bp probe to make a longer 33-bp probe and repeated the EMSA experiment, again KdpE was found to be able to bind successfully with this longer probe (Figure 3). Furthermore, a competition EMSA was carried out with increasing concentrations of unlabeled 33-bp probe along with 300-bp 6-FAM-labeled probe in the reaction mixture. The unlabeled 33-bp probe showed specific binding with KdpE and competed well with the labeled probe as revealed in the gel (Figure 3A).

**Figure 4:**
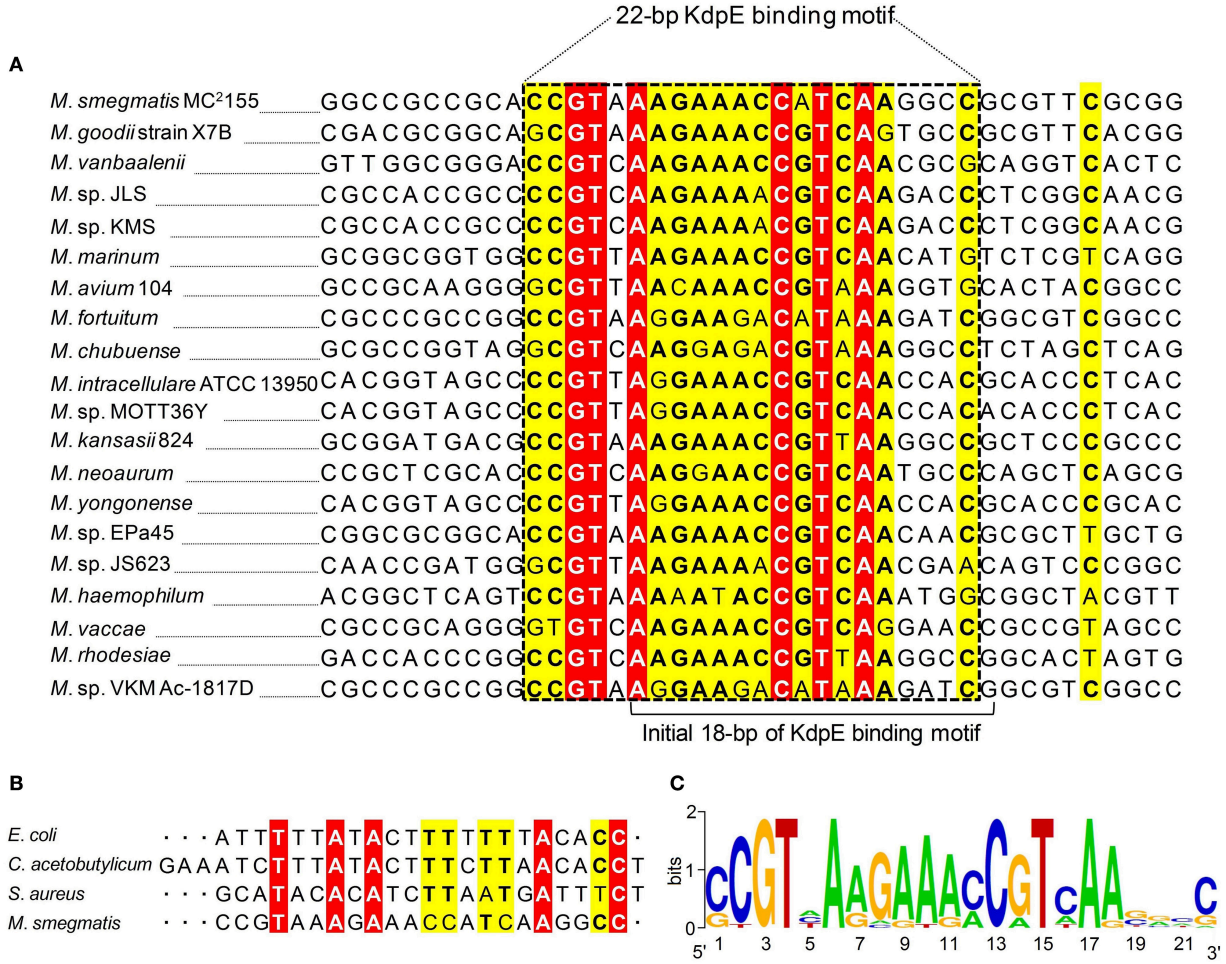
**Multiple DNA sequences alignments of KdpE binding motifs from different mycobacterial species. (A)** The homology of minimum 22-bp binding sequence was searched in different species of genus *Mycobacterium*. Highly similar nucleotides are shown in white letters highlighted in red background, less similar nucleotides in black bold letters highlighted in yellow, while dissimilar nucleotides in black letters in white background. **(B)** KdpE binding sequences found in different bacterial species; *E. coli* (Sugiura et al., 1992), *C. acetobutylicum* (Behrens and Duerre, 2000), *S. aureus* (Xue et al., 2011) and *M. smegmatis* (present study). **(C)** The DNA logo of DNA motifs required for efficient binding of KdpE protein to the P*kdpF* based on DNA homology among different mycobacterial species. The logo was prepared using WebLogo (Crooks et al., 2004).

To attain the minimum sequence required for the KdpE binding, few nucleotides were sequentially removed from both ends of the effective 33-bp probe (Figure 3B) to see if shorter fragments still retained the binding ability with KdpE. In this aspect, a sequence of 28-bp but not any other shorter sequence was found to retain successful binding with KdpE (Figure 3C). We then further removed nucleotides from 3′-end of the 28-bp sequence to generate a series of shorter fragments (Figure 3B). Finally, a minimum of 22-bp sequence (5′-CCGTAAAGAAACCATCAAGGCC-3′) with strong binding ability with KdpE was identified (Figure 3D). This 22-bp sequence contained five additional nucleotides at the 5′-end and one nucleotide less from the 3′-end of the original 18-bp foot print sequence. This newly determined 22-bp motif was found to be highly conserved in many mycobacterial species (Figure 4).

### TSS of the *kdpFABC* operon

**Figure 5:**
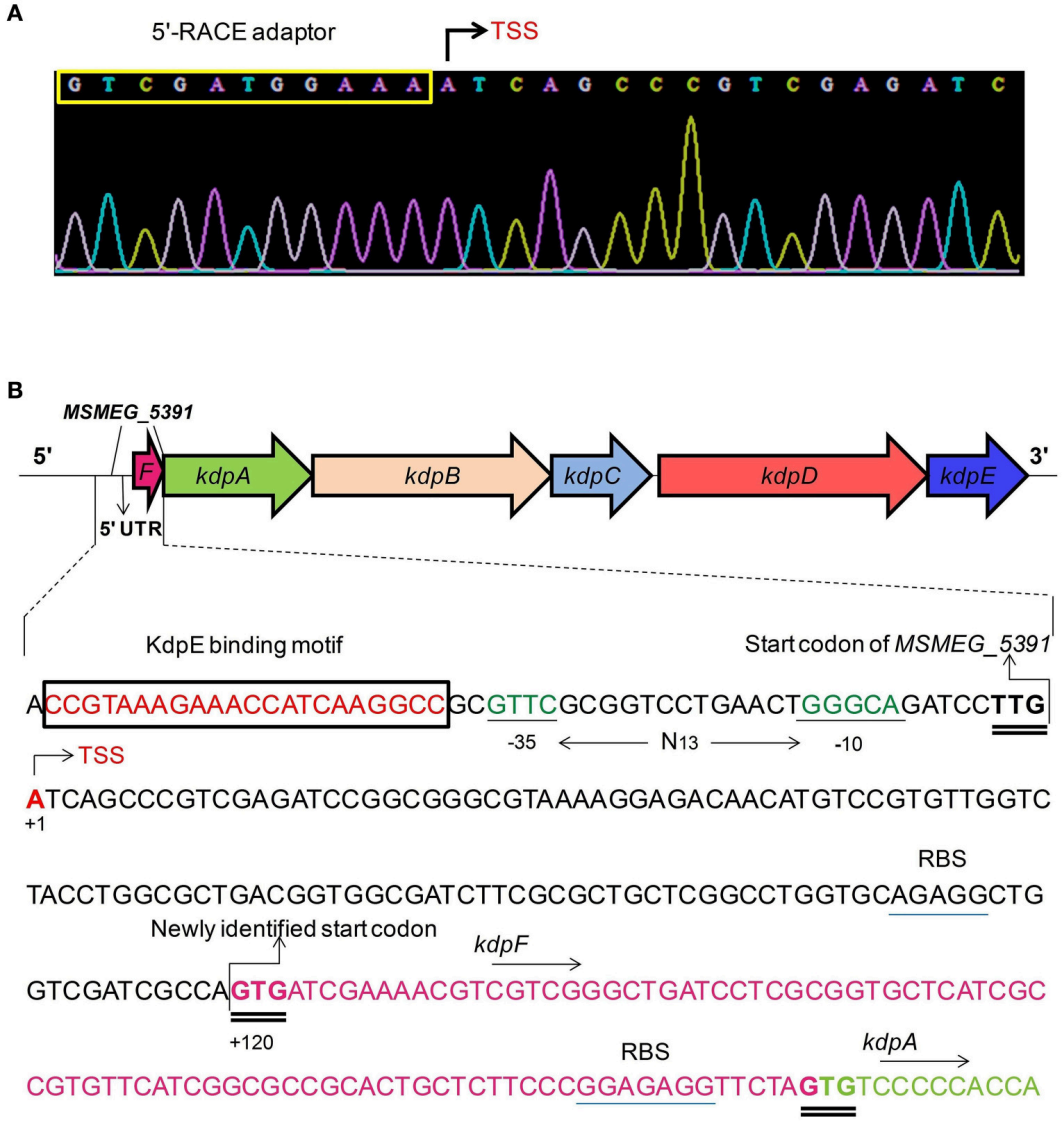
**Mapping of TSS and characterization of the P***kdpF***. (A)** The 5′-RACE adaptor sequence (boxed in yellow) along with the transcript sequence after DNA sequencing. The TSS is marked with a bent arrow. **(B)** Fully characterized P*kdpF* sequence, where the KdpE binding sequence is shown in red nucleotides boxed in black, the SigF-dependent promoter consensus −10 and −35 regions shown in dark green, the TSS nucleotide shown with red letter marked by a bent arrow. All potential start codons are double underlined, with the newly identified start codon indicated at the +120 position. The candidate *kdpF* gene is shown in magenta letters. The sequence of *MSMEG_5391* containing a 5′-UTR region (120 bp) of *kdpFABC* and the newly determined *kdpF* coding sequence (87-bp) is shown overlapped with the start codon of *kdpA*. The possible Shine-Dalgarno sequences in RBS are underlined with blue line.

**Figure 6:**
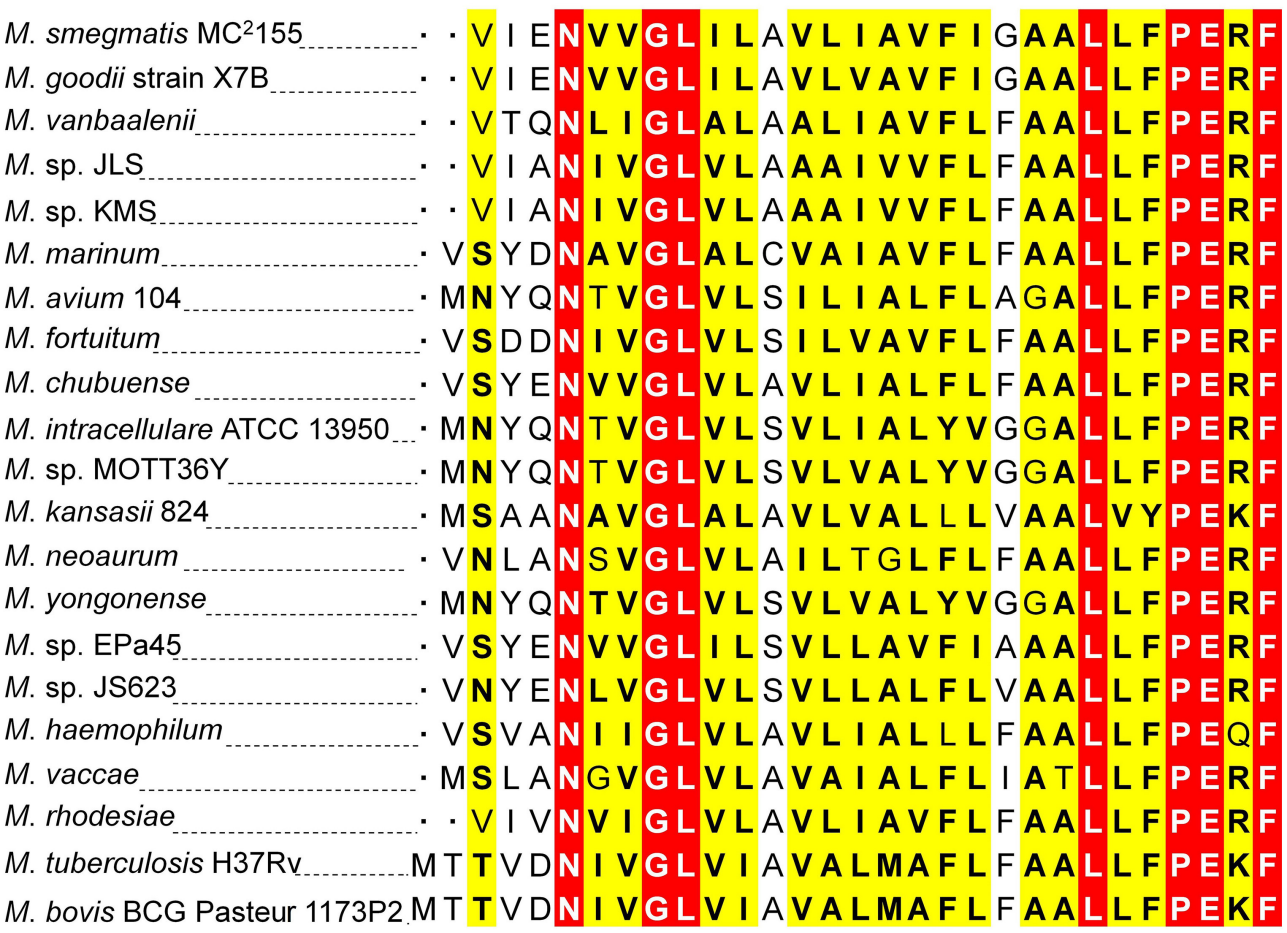
**Protein sequence homology of KdpF of ***M. smegmatis*** with other mycobacterial species**. The DNA sequence of *kdpF* in *M. smegmatis* was determined based on 5′-RACE and subsequent β-galactosidase assays. Its homologs were searched in other mycobacterial species in the upstream region of *kdpA*. The KdpF protein sequences of high similarity are shown in white letters highlighted in red background, low similarity in black bold letters highlighted in yellow background, and dissimilar residues in black letters in white background.

**Figure 7:**
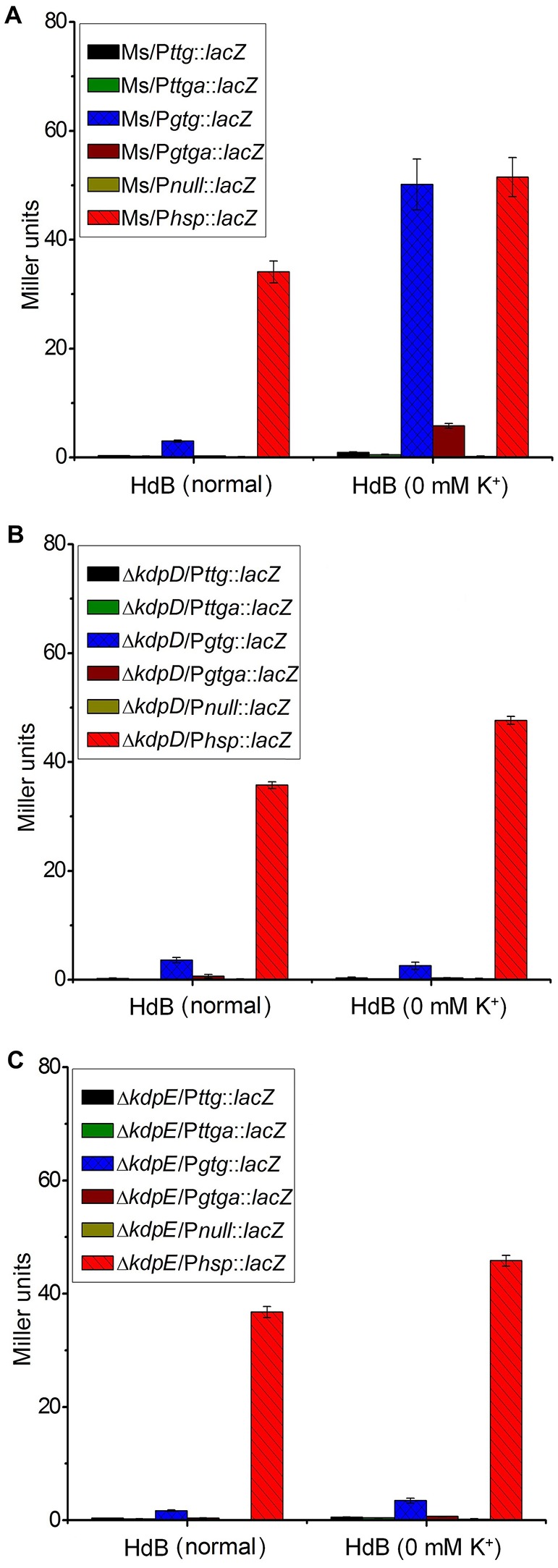
**Induction of ***kdpF*** under K^**+**^ limiting conditions**. β-galactosidase assay was performed to determine functionality of P*kdpF*, translation start codon of *MSMEG_5391* (TTG) as well as newly identified translation start codon of *kdpF* (GTG). Promoter regions were fused with promoter less-*lacZ* gene (in frame and out of frame) in pMV261 to generate the P*ttg*::*lacZ*, P*ttga*::*lacZ*, P*gtg*::*lacZ*, and P*gtga*::*lacZ* plasmids. Plasmids were separately transferred into wild type **(A)**, Δ*kdpD* **(B)**, and Δ*kdpE* **(C)** to determine regulation of P*kdpF* by KdpD/KdpE TCS. For positive control, P*hsp* was fused with the *lacZ* gene (P*hsp*::*lacZ*) and for the negative control promoter less-*lacZ* plasmid (P*null*::*lacZ*) was used. Strains were grown in HdB broth under K^+^ normal and limiting conditions (0 mM K^+^) and β-galactosidase activity was determined to investigate promoter functionality.

Two 5′-RACE cDNA libraries (in the presence and absence of K^+^) were constructed from the wild type strain. Amplification of expected RACE inner fragment was only possible when RT-PCR was performed using the K^+^ limiting library, which revealed a sharp band on agarose gel, whereas no such band was detected using the normal K^+^ library (data not shown). First nucleotide base identified next to the 5′-RACE adaptor was an adenosine residue (A) that was considered as the TSS of the *kdpFABC* operon (Figure 5A). Unexpectedly, this TSS was found within the coding sequence of *MSMEG_5391* at the +4-bp position. Hence, the ORF of the candidate *kdpF* was supposed to be present within this region. DNA sequence analysis revealed another possible start codon GTG located at the +120-bp position of TSS (Figure 5B). The candidate *kdpF* gene (87-bp) shared significant homology to the *kdpF* of *M. tuberculosis* (93-bp). Furthermore, this candidate *kdpF* was aligned to the upstream regions of *kdpA* in various mycobacterial species. Almost all species in which KdpE binding sequence was conserved, contained a *kdpF* gene which shares significant identity to the *kdpF* of *M. smegmatis* and *M. tuberculosis* (Figure 6). Further, β-galactosidase assay (with and without frame shift) was conducted to verify candidate *kdpF* as a functional gene of *kdpFABC* operon. Together, all data showed that the 87-bp ORF is the part of *kdpFABC* operon which was successfully induced under low K^+^ conditions (Figure 7). Since the start codon of *MSMEG_5391* was not functional, this confirmed that the previously identified hypothetical protein (MSMEG_5391) was very likely to be annotated incorrectly. In contrast, this genomic locus (*MSMEG_5391*) was believed to contain a long 5′-UTR and a functional *kdpF* gene.

### Effects of the salts on the expression of *kdpFABC*

A series of β-galactosidase assays was performed to investigate potential stimuli for the transcriptional induction of the *kdpFABC* operon and its regulation by the KdpD/KdpE TCS. For this purpose, different promoter sequences were fused with *lacZ* (Figure S7). Under K^+^ non-limiting condition, no β-galactosidase activity was observed in wild type strains, except for some basal level activity in the Ms/P*gtg*::*lacZ*, whereas under K^+^ limiting condition, very high β-galactosidase activity was observed for Ms/P*gtg*::*lacZ* compared to all other strains (Figure 7A). There was no β-galactosidase activity observed in the Δ*kdpD* and Δ*kdpE* in both, normal and 0 mM K^+^ HdB (Figures 7B,C). These results also supported our previous RT-qPCR results in which deletion of *kdpD*/*kdpE* genes resulted in silencing of the *kdpFABC* operon.

**Figure 8:**
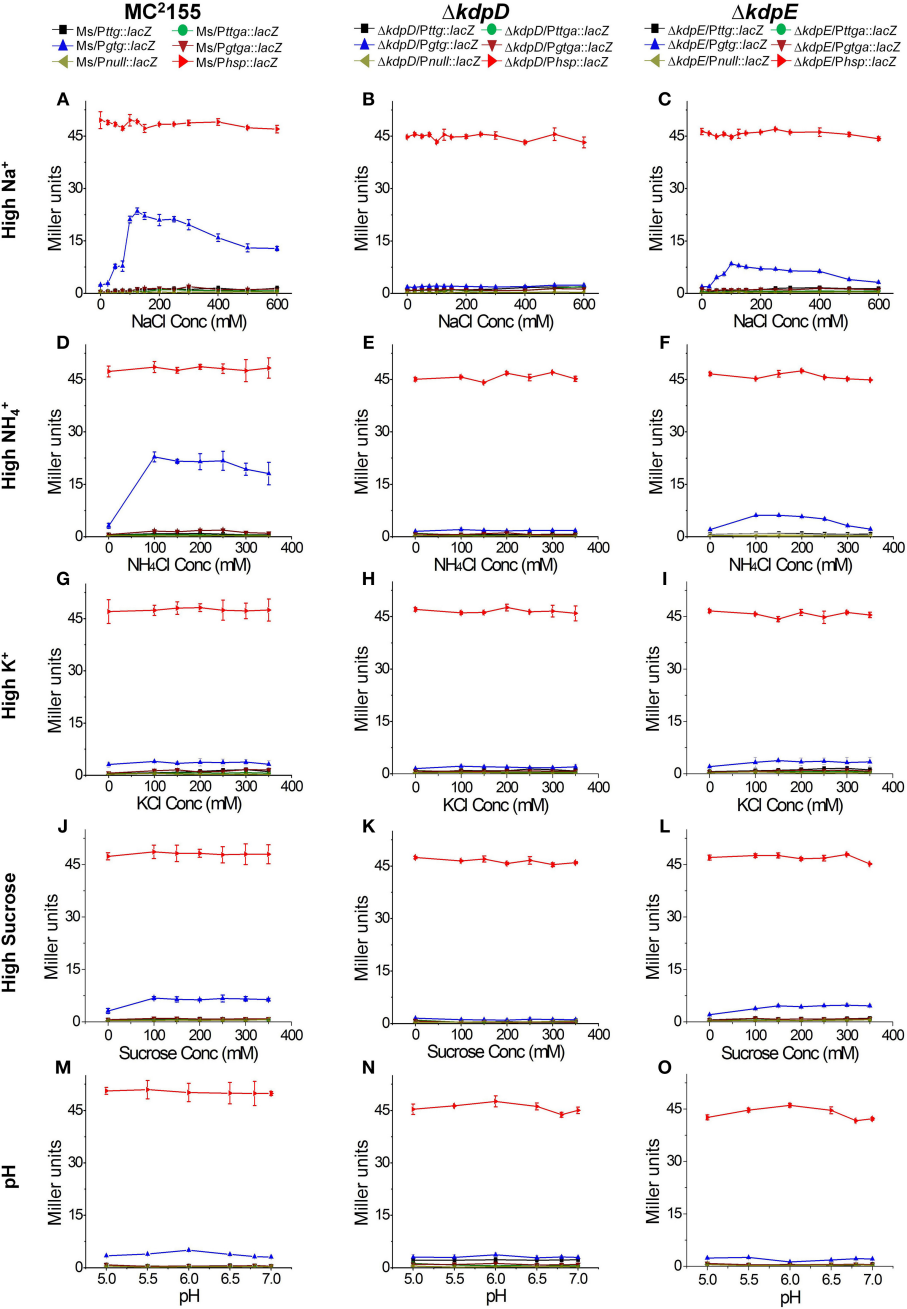
**Promoter functionality and effect of different stimuli on ***kdpFABC*** transcription**. The strains used are described in Figure 7. Strains were grown in 7H9 broth under different concentrations of NaCl **(A–C)**, NH_4_Cl **(D–F)**, KCl **(G–I)**, sucrose **(J–L)**, and pH-values **(M–O)**. β-galactosidase activities were determined to investigate promoter functionality and expression of *kdpFABC*.

In order to further explore the potential signals other than low K^+^ condition that could turn on *kdpFABC* transcription in *M. smegmatis*, all strains previously constructed for β-galactosidase activity (Table 1) were grown in 7H9 medium with different concentrations of NaCl, NH_4_Cl, KCl, and sucrose as well as under different pH-values. In case of 7H9 medium alone, none of the wild type derivatives exhibited noticeable β-galactosidase activity. However, with increasing concentrations of NaCl and NH_4_Cl, β-galactosidase activity of Ms/P*gtg*::*lacZ* was increased. On the other hand, minor and no activity was observed in the case of increasing concentrations of sucrose and KCl, respectively. Moreover, no activity change was observed under different pH-values. The derivatives of Δ*kdpD* showed no β-galactosidase activity in all above mentioned conditions, while in the Δ*kdpE* derivatives, only Δ*kdpE*/P*gtg*::*lacZ* showed slight activity with increasing NaCl or NH_4_Cl concentrations that was considerably lower than Ms/P*gtg*::*lacZ* under the same conditions (Figure 8).

### Requirement of KdpE for the normal growth of *M. smegmatis*

**Figure 9:**
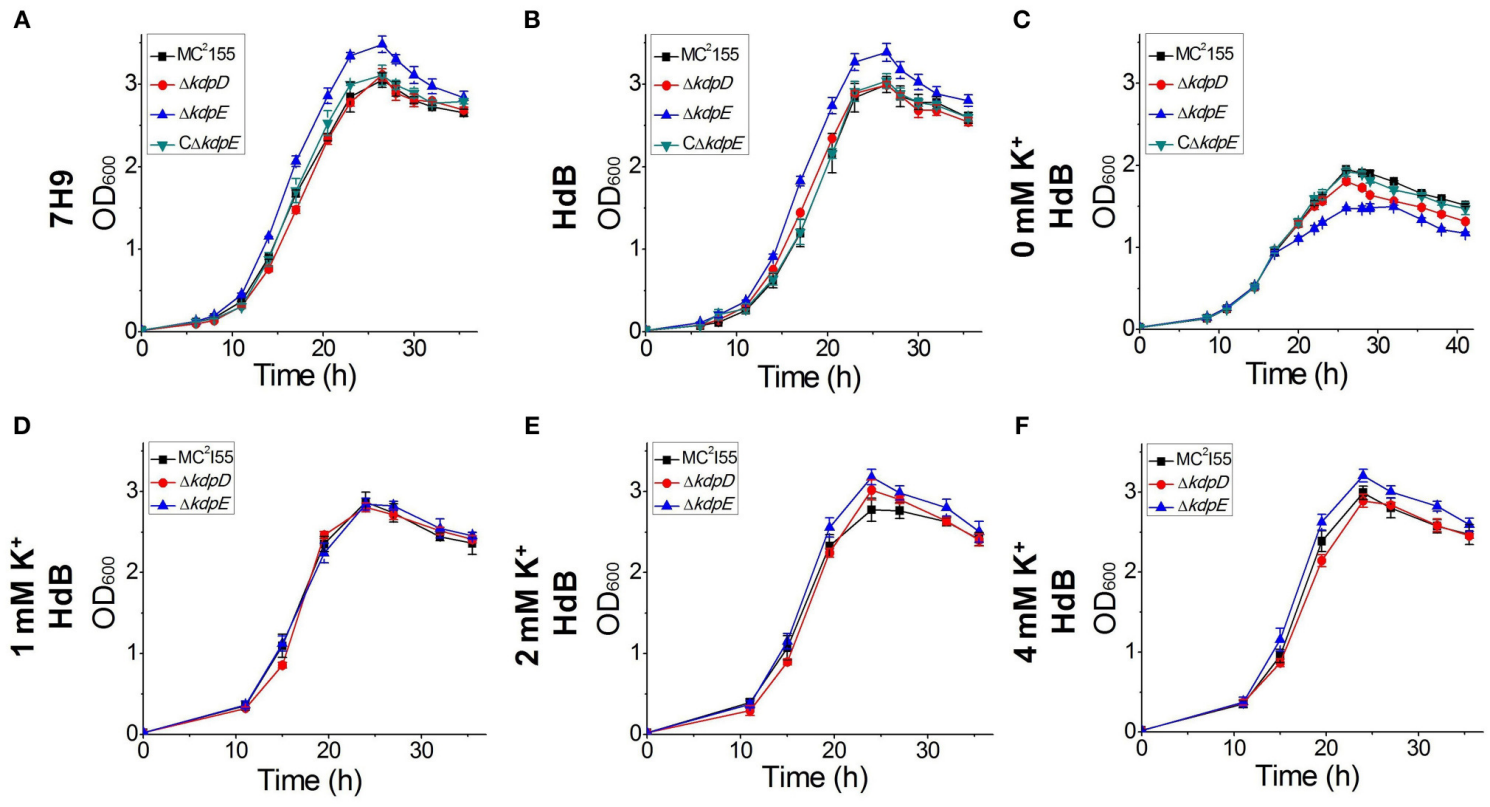
**Growth curves of wild type MC^**2**^155 and its derivative strains under different concentrations of K^**+**^ in HdB medium**. Bacterial growth curves were generated by growing all bacterial strains including wild type MC^2^155, Δ*kdpD*, Δ*kdpE*, and CΔ*kdpE*, in 7H9 and HdB broth (with and without K^+^). Overnight cultures of all the strains were used to inoculate fresh media at a starting OD_600_ of 0.02. Each experiment was performed in triplicate with mean values shown in the graph. Cultures were incubated at 37°C under stirring condition for 40 h. OD_600_ was determined by sampling cultures in every 3 h. **(A)** Growth experiment for *M. smegmatis* and its derivatives was studied in 7H9 broth. Similarly, growth curves of *M. smegmatis* and its derivatives were also generated in HdB medium under different concentrations of K^+^ **(B** = normal, **C** = 0 mM, **D** = 1 mM, **E** = 2 mM, **F** = 4 mM**)**.

The growth of wild type as well as Δ*kdpD* and Δ*kdpE* mutant strains was investigated in two different growth media (7H9 and HdB). The growth patterns of wild type and Δ*kdpD* were mostly similar without much difference, whereas Δ*kdpE* showed a slightly faster growth rate (Figures 9A,B).

Medium K^+^ concentrations influenced the growth rates of the wild type and mutant strains. At 0 mM K^+^ in HdB medium, the growth of all strains started with a relatively longer lag phase but shorter logarithmic phase in comparison to the growth in normal K^+^ condition. Besides, a noticeable defect was observed for the growth of Δ*kdpE* (Figure 9C). At 1 mM K^+^ concentration, all strains grew equally well with rates similar to those of the wild type in 7H9 and HdB media (Figure 9D). Increasing K^+^ up to 2 mM or above, the growth rates of Δ*kdpE* slightly raised as compared to the wild type and Δ*kdpD* (Figures 9E,F). Furthermore, to confirm whether this altered growth behavior of Δ*kdpE* strain was solely associated with *kdpE* deletion, we repeated the growth experiments in 7H9, HdB media (with normal K^+^) and HdB (with 0 mM K^+^) using the complementary strain of Δ*kdpE*. The growth curve of the CΔ*kdpE* strain was found to be similar to the wild type strain (Figures 9A–C), confirming that the altered growth behavior of Δ*kdpE* was attributed to *kdpE* deletion.

Attempts were also made to detect responses of Δ*kdpD* and Δ*kdpE* to different stress conditions. For this, bacterial strains were exposed to various stress conditions for different time intervals and then plated onto 7H10 medium. No significant differences were observed in the growth patterns of wild type and mutant strains under most of the stresses studied. However, under the heat shock stress of 50°C for 5 h, Δ*kdpE* appeared to be slightly resistant. On the contrary, under hypo-osmotic stress condition in H_2_O and acidic pH 4.0 in citrate phosphate buffer for 5 h, Δ*kdpE* appeared to be slightly sensitive (Table S2). Overall these data demonstrated that KdpE was required for the normal growth of *M. smegmatis*.

## Discussion

K^+^ plays many physiological roles in bacteria and its sufficient supply is essential for the pathogenicity of various species including *M. tuberculosis* (Salina et al., 2014). Upon K^+^ deficiency, bacterial cells of *M. tuberculosis* entered into a dormant non-culturable state but could be revitalized to culturable state once the availability of K^+^ was restored. Moreover, those non-culturable cells acquired resistance to the cell wall-targeting antimicrobials. Hence this resistance dormancy may raise the probability of latent tuberculosis (Salina et al., 2014). Regarding the important roles of K^+^ in mycobacterial physiology and pathogenicity, elucidation about K^+^ uptake and its regulatory mechanism would enable us to control mycobacterial infections in a more efficient way.

**Figure 10:**
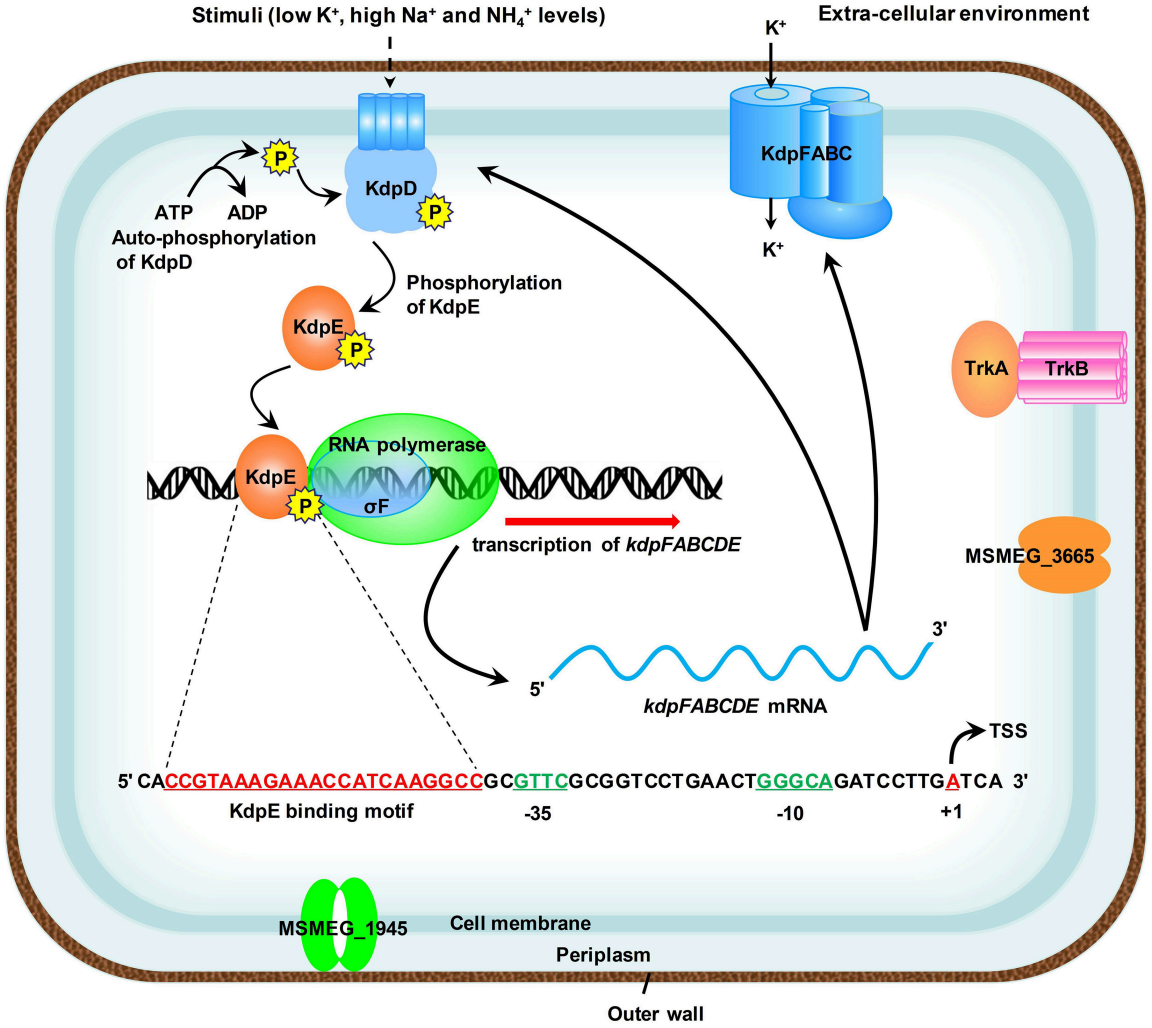
**Proposed schematic representation of the induction and transcription of ***kdpFABC*** operon in ***M. smegmatis*****. Membrane bound KdpD senses stimuli such as low K^+^, high Na^+^, and ${\text{NH}}_{4}^{+}$ concentrations of the medium. KdpE acts as a transcriptional regulator and is phosphorylated by KdpD to positively regulate the transcription of *kdpFABC* operon along with SigF-dependent RNA polymerase in a cooperative way. The 22-bp KdpE binding sequence is shown in red and SigF-dependent promoter consensus −35 and −10 regions are shown in green. The TSS nucleotide is shown in red with bend arrow. The Trk K^+^ uptake system and other possible channels are also shown in the diagram.

The species of genus *Mycobacterium* relies mainly on the Trk and Kdp systems for K^+^ uptake (Cholo et al., 2008). The Kdp-ATPase is regulated by KdpD/KdpE TCS, which was found to be functional in *M. tuberculosis* (Agrawal and Saini, 2014) and the bacterial strain became hyper-virulent upon its deletion (Parish et al., 2003). However, noticeable variations in the genetic organization of *kdpFABC* and *kdpDE* operons were observed within the genus *Mycobacterium* (Steyn et al., 2003; Cholo et al., 2008; Agrawal and Saini, 2014) (Figure S8). Some of the pathogenic species of genus *Mycobacterium* such as *M. leprae* and *M. ulcerans* are even deprived of Kdp-ATPase system (Cholo et al., 2008). Since different species of the genus *Mycobacterium* pose serious threats to a wide variety of organisms, the mechanism of K^+^ uptake within this genus needs to be elucidated in detail in order to cope with their pathogenic potential. To date, regulation of Kdp-ATPase has not been thoroughly investigated in *M. smegmatis*, a model species that has been used as a surrogate for pathogenic mycobacterial species. This manuscript focused on the regulation of *kdpFABC* operon by KdpD/KdpE TCS and, for the first time, proposed a detailed regulatory mechanism in *M. smegmatis* (Figure 10). These findings shall provide a foundation to further explore the mechanism of K^+^ uptake by Kdp-ATPase in other pathogenic species of genus *Mycobacterium*.

In *M. smegmatis*, it was found that the expression of *kdpFABC* operon was regulated by a KdpD/KdpE TCS that was induced under K^+^ limiting condition. Similar phenomenon was also reported in other bacteria (Altendorf et al., 1992; Frymier et al., 1997; Treuner-Lange et al., 1997). The KdpE binding sequence in P*kdpF* was found to be well-conserved in various mycobacterial species (Figure 4). However, no such sequence similarity could be detected in *M*. *tuberculosis*. Our results support the previous study that attempted to reveal binding of KdpE~P to the 192-bp intergenic region between *kdpFABC* and *kdpDE* in *M. tuberculosis*, although no such binding was observed (Agrawal and Saini, 2014). Contrary to *M. tuberculosis*, the direct binding of KdpE in P*kdpF* was confidentially observed in *M*. *smegmatis* in the present study.

Initially, candidate P*kdpF* (upstream of *MSMEG_5391*) was used for EMSA and DNase-I foot printing studies, considering *MSMEG_5391* as a part of the *kdp* operon. However, the newly identified TSS was the fourth nucleotide of *MSMEG_5391* hence, we believed that the original annotation of ORF (*MSMEG_5391*) might be inaccurate since the first three nucleotides of this frame were not the part of mRNA. The downward sequence of *MSMEG_5391* was therefore searched for another potential ORF of *kdpF* gene. The sequence similarity of this newly identified *kdpF* of *M. smegmatis* with those of other species in genus *Mycobacterium* was noteworthy, although in many mycobacterial species, *kdpF* is still un-annotated. Our findings provide a strong foundation to identify and characterize *kdpF* in other species of genus *Mycobacterium*. Previously, KdpF was reported as a regulatory peptide (Gannoun-Zaki et al., 2013, 2014), hence its role under stress conditions needs further investigations in *M. smegmatis*.

The *sigF* loci in *M. smegmatis* encoded an alternative stress-response sigma factor F (SigF) (Gebhard et al., 2008). Previously, SigF-dependent promoters were first identified in *M. tuberculosis* (Rodrigue et al., 2007), and also characterized in *M. smegmatis* (Gebhard et al., 2008) in later studies. A GTTT-N_(15−17)_-GGGTA motif was predicted as SigF-dependent promoter consensus after inspecting promoter sequences of numerous SigF-regulated genes through bioinformatics analysis by the same group (Hümpel et al., 2010). In our study, we found that the −10 and −35 consensus sequences of P*kdpF* were highly similar to numerous SigF-regulated sequences so far identified, but with a smaller *N* numbers (GTTC-N_(13)_-GGGCA). In the previous study, P*kdpF* was not identified as a SigF-regulated promoter (Hümpel et al., 2010). This difference might be due to two reasons. First, the previous conclusion was based on different growth phases, whereas extremely low K^+^ concentration or high salt stress are required for the activation of *kdpFABC* transcription. Since their microarray study was performed under a non-inducible condition, *kdpFABC* might not be expressed at all. Second, different criteria were adopted in choosing spacing between the −10 and −35 regions of the SigF-dependent promoter consensus.

Ionic strength has been reported as stimulus for the induction of Kdp system. For instance, the Kdp system was found to be stimulated at high Na^+^ and ${\text{NH}}_{4}^{+}$ concentrations in *E. coli* (Jung et al., 2001; Hamann et al., 2008; Epstein, 2016). Similar to these findings, Kdp-ATPase of *M. smegmatis* was also induced under high concentrations of Na^+^ and ${\text{NH}}_{4}^{+}$ salts. Results of our RT-qPCR data further verified the induction of Kdp system under Na^+^ and ${\text{NH}}_{4}^{+}$ salts stresses (Figure S9). In the case of *M. tuberculosis*, bacterial systems related to K^+^ uptake were induced at acidic pH (Cholo et al., 2015). Similarly, in *E. coli*, the expression of *kdpFABC* also altered due to the change in the medium pH (Asha and Gowrishankar, 1993). However, we found that the Kdp-ATPase system of *M. smegmatis* was not induced by medium pH change.

In our study on *M. smegmatis*, a single transcript of *kdpFABCDE* was observed under K^+^ limiting condition. In *E. coli*, however, it was proposed that some long transcripts were made to contain transcripts of both operons but most of the transcripts stopped before the start of *kdpD* (Altendorf et al., 1992). The single transcript (*kdpZYABCXDE*) in K^+^ limiting condition was reported in *Clostridium acetobutylicum* (Treuner-Lange et al., 1997) and (*kdpABC*-*DE*) *in Acinetobacter baumannii* (Samir et al., 2016). In case of *M. smegmatis*, it could be proposed that under low K^+^ concentration, both operons (*kdpFABC* and *kdpDE*) were co-transcribed at least to some extent from P*kdpF*, whereas under normal K^+^ condition, *kdpDE* was transcribed to very basal level and this transcription was initiated from P*kdpD*.

Kdp-ATPase and its regulatory KdpD/KdpE TCS have been reported in many bacterial species including *E. coli, S. aureus, C. acetobutylicum, M. tuberculosis, S. typhimurium, Leptospira interrogans, Alicyclobacillus acidocaldarius*, and *A. baumannii* for its role in K^+^ uptake (Ballal et al., 2007; Xue et al., 2011; Matsunaga and Coutinho, 2012; Samir et al., 2016). In bacterial species such as *E. coli, L. interrogans*, and *A. baumannii*, KdpE was highly expressed in order to positively regulate expression of *kdpFABC* operon under low K^+^ concentrations (Polarek et al., 1992; Matsunaga and Coutinho, 2012; Samir et al., 2016) except in *S. aureus* where KdpE negatively regulated *kdpFABC* expression (Xue et al., 2011). In *M. tuberculosis*, KdpD/KdpE TCS was found to be functional (Agrawal and Saini, 2014) and it was reported that KdpD interacted with two membrane lipoproteins (LprF and LprJ) to modulate expression of *kdpFABC* controlled by KdpE under low K^+^ concentrations (Steyn et al., 2003). At present, KdpE binding sequence in the P*kdpF* has only been identified in four bacterial species including *E. coli* (Sugiura et al., 1992), *C. acetobutylicum* (Behrens and Duerre, 2000), *S. aureus* (Xue et al., 2011), and *M. smegmatis* (current study). The *C. acetobutylicum* KdpE binding motif shared significant sequence similarity with that of *E. coli* (23 bp T-rich). However, *M. smegmatis* KdpE binding motif (22 bp A-rich) did not show noticeable sequence similarity with any other previously reported KdpE binding motif (Figure 4B).

In *M. smegmatis*, bioinformatics study revealed another constitutively expressed system for K^+^ uptake, namely the Trk system comprised TrkA (MSMEG_2771) and TrkB (MSMEG_2769) subunits. In addition, TrkA domain-containing protein (MSMEG_3665) and ion channel membrane protein (MSMEG_1945) were found as possible K^+^ channels. Expression of these genes was down-regulated in K^+^ limiting (0 mM K^+^) condition when compared to K^+^ non-limiting conditions (normal K^+^) in wild type and mutant strains (Figure S10). These results indicated that under K^+^ limiting condition, the Kdp-ATPase appeared to be functional to scavenge K^+^ as compared to other K^+^ systems.

It can be concluded that in *M. smegmatis*, Kdp-ATPase plays a vital role in the bacterial physiology during K^+^ limitation. The system is positively regulated by KdpD/KdpE TCS and triggered for osmo-regulation during low K^+^ and salt stress. The regulation of *kdpFABC* operon via KdpD/KdpE TCS appeared to be dependent upon SigF. Based on growth discrepancies of Δ*kdpD* and Δ*kdpE*, it can be proposed that some other histidine kinases may also activate KdpE, which in return regulates genes other than *kdpFABC*. However, a detailed study is required to verify this hypothesis.

For the first time, DNA sequence required for successful binding of KdpE with P*kdpF* was identified in genus *Mycobacterium*. Based on sequence similarity of the DNA motif, the binding of KdpE protein to positively regulate Kdp-ATPase can be expected in other mycobacterial species such as *M. avium, M. fortuitum, M. marinum*, and *M. chubuense*. On the other hand, a different mechanism might be speculated for the regulation of Kdp-ATPase in *M. tuberculosis* and *M. bovis*, which lack this KdpE-binding DNA motif and disclose different genomic organization for the *kdpFABC* and *kdpDE* operons.

In the present manuscript, we also observed a comparatively long 5′-UTR region in the transcript of *kdpFABC* for *M. smegmatis*. It would be worthy of further studies to see whether this 5′-UTR region exhibits some kind of regulatory role for the translation of Kdp-ATPase transporter.

## Author contributions

MKA performed most of the experiments and made most of the data evaluation. XLi, QT, XLiu, FC, JX and MA participated in partial experiments and interpretation of the data. MKA, QT, and JH conceived the study and drafted the manuscript. JH, SC, and MA revised the manuscript. All authors read and approved the final manuscript.

### Conflict of interest statement

The authors declare that the research was conducted in the absence of any commercial or financial relationships that could be construed as a potential conflict of interest.

## Supplementary material

Figure S1 **Schematic diagram of ***kdpD*** gene knock-out procedure in ***M. smegmatis*** MC^**2**^155**. The *kdpD* mutation was carried out by the method of homologous recombination as described earlier (Yang et al., 2012). The 800-bp upstream and downstream regions flanking the target *kdpD* gene were amplified by PCR, purified and transferred into pMD19-T for sequence verification. These fragments were cloned into pMind vector (containing a hygromycin marker) at specific cloning sites. For Δ*kdpD*, hygromycin marker was excised from vector to create unmarked mutation. The resulting plasmid containing upstream and downstream regions was digested with PacI enzyme to insert the *sacB*-*lacZ* cassette from pGOAL17. This final suicide delivery vector was then electroporated into *M. smegmatis* MC^2^155 competent cells after 5 min direct exposure to UV radiation, and plated on 7H10 medium containing kanamycin (30 μg/mL) and X-gal (50 μg/mL) for single crossover (SCO). The obtained SCO colonies were allowed to grow in 7H9 broth without antibiotics to facilitate double crossover (DCO). One hundred microliters of culture was then plated on 7H10 medium containing 10% sucrose and X-gal (50 μg/mL). Resulting white colonies were tested for kanamycin susceptibility.

Figure S2 **Schematic diagram of ***kdpE*** gene knock-out procedure in ***M. smegmatis*** MC^**2**^155**. The *kdpE* knock-out was performed by the method of homologous recombination as described earlier (Yang et al., 2012). The 800-bp upstream and downstream regions flanking the target *kdpE* gene were amplified by PCR, purified and transferred into pMD19-T for sequence verification. These fragments were cloned into pMind vector (containing a hygromycin marker) at specific cloning sites. For *kdpE* gene, marked mutation method was used. The resulting plasmid containing upstream and downstream regions was digested with PacI enzyme to insert the *sacB*-*lacZ* cassette from pGOAL17. This final suicide delivery vector was then electroporated into *M. smegmatis* MC^2^155 competent cells after 5 min direct exposure to UV radiation and plated on 7H10 medium containing kanamycin (30 μg/mL), X-gal (50 μg/mL), and hygromycin B (100 μg/mL) for SCO. The obtained SCO colonies were allowed to grow in 7H9 broth without antibiotics to facilitate DCO and 100 μl culture was spread over 7H10 medium containing 10% sucrose, X-gal (50 μg/mL) and hygromycin B (100 μg/mL). Resulting white colonies were tested for kanamycin susceptibility.

Figure S3 **Construction of complementary strain of ***kdpE*****. Promoter region (P*kdpD*) was amplified by PCR using CP-D-F and CP-D-R primers (Table S1) and confirmed by sequencing. The *hsp60* promoter (P*hsp*) in expression plasmid pMV261 (Yang et al., 2012) was replaced by P*kdpD* followed by insertion of *kdpE* gene that was amplified by primers C-kdpE-F and C-kdpE-R (Table S1). The resulting recombinant plasmid was confirmed by restriction endonucleases and PCR amplification. Plasmid was then electroporated into Δ*kdpE* competent cells. Resulting colonies were plated on 7H10 with kanamycin (30 μg/mL), and confirmed by RT-PCR for the expression of *kdpE* gene in CΔ*kdpE* strain.

Figure S4 **Heterologous expression of KdpE in ***E. coli*** BL21(DE3)-pET28-***kdpE*****. KdpE protein from *M. smegmatis* genome was amplified and cloned into pET28a(+) to obtain expression plasmid pET28-*kdpE* that was transferred to *E. coli* BL21(DE3) to obtain the over-expression strain BL21(DE3)-pET28-*kdpE*. Isopropyl β-D-1-thiogalactopyranoside (IPTG) was added to the culture when OD_600_ reached to 0.5 and incubated at 28°C for 12 h for protein expression. Cells were harvested and re-suspended in lysis buffer (20 mM imidazole, 20 mM tris-base, 1 M NaCl) and lysed by ultrasonication. Crude cell lysate was centrifuged to remove debris and supernatant was used to purify 6 × His KdpE by using affinity column (Ni-NTA, GenScript). Cell lysate and purified KdpE protein were mixed with 2 × SDS loading buffer, boiled and centrifuged to run on 12% SDS PAGE. Lane 1: cell lysate, lane 2: protein marker, lane 3: purified KdpE protein (28.5 kDa).

Figure S5 **Co-transcription of ***kdpFABC*** and ***kdpDE*** operons under K^**+**^ limiting conditions**. Genomic organization of the *kdpFABC* and *kdpDE* genes and small regions amplified in RT-PCR are shown by numbered short lines. Amplified PCR products obtained from K^+^ limiting cDNA were separated on 1% agarose gel. CK^+^ denotes amplification of a segment of *sigA* which was used as positive control, while CK^−^ denotes PCR mixture without cDNA which was used as negative control.

Figure S6 **Promoter functionality β-galactosidase assays. (A)** About 500 bp upstream region of *MSMEG_5391* was taken as candidate promoter (P*5391*) and fused with promoter less *lacZ* gene to construct plasmid pMV261-P*5391*::*lacZ*. Newly constructed plasmid pMV261-P*5391*::*lacZ* was transformed into wild type and Δ*kdpE*. β-galactosidase activity was determined under normal and 0 mM K^+^ condition. **(B)** To study induction of promoter of *kdpDE* (P*kdpD*) under normal and 0 mM K^+^, about 500 bp upstream region of *kdpDE* operon was fused with promoter less *lacZ* and the newly constructed plasmid pMV261-P*kdpD*::*lacZ* was transformed into wild type and Δ*kdpE*. β-galactosidase activity was determined under normal and 0 mM K^+^ condition. Data represents the averages of biological triplicates. Error bars indicate standard deviation.

Figure S7 **Design of promoter::***lacZ*** vectors constructed for the identification of exact promoter sequence and ***kdpF*** identification. (A)** The organization of annotated *MSMEG_5391* with nucleotide sequence is shown in green. The start and termination codons of annotated *MSMEG_5391* are boxed in black. **(B)** The 300 bp candidate promoter region upstream of annotated *MSMEG_5391* gene with the start codon of *MSMEG_5391* (TTG) fused with *lacZ*. **(C)** The 300 bp candidate promoter region upstream of *MSMEG_5391* with the start codon of *MSMEG_5391* plus one extra nucleotide (TTGA) to induce a frame-shifted *lacZ*. **(D)** The organization of newly proposed *kdpF* with nucleotide sequence is shown in magenta. The start and termination codons of newly proposed *kdpF* are boxed in black. **(E)** The P*kdpF* plus 5′-UTR of newly proposed *kdpF* with start codon (GTG) fused with *lacZ*. **(F)** The P*kdpF* plus 5′-UTR of newly proposed *kdpF* with start codon plus one extra nucleotide (GTGA) to induce frame shifted *lacZ*.

Figure S8 **Organization of ***kdpFABC*** and ***kdpDE*** operons in different mycobacterial species**. The positioning of *kdpFABC* and *kdpDE* operons in different mycobacterial species are shown in gene locus tags. Colored arrows showed different genes, with *kdpF* in magenta, *kdpA* in light green, *kdpB* in light orange, *kdpC* in light blue, *kdpD* in red, and *kdpE* in dark blue. Arrow head represents gene direction. Most of the *kdpF* sequences shown are identified in this study by sequence homology, and locus tags of *kdpF* are designated only to those species in which *kdpF* was previously and correctly annotated by NCBI/KEGG. In all species of mycobacteria that are analyzed in this study, the *kdpDE* operon is located adjacent to *kdpFABC* operon with the exception of *M. chubuense*, in which two operons are distantly located in same orientation. In case of *M. chubuense*, two slanted red lines indicate that two operons are not adjacent to each other.

Figure S9 **Induction of ***kdpF*** and ***kdpDE*** genes under osmotic stress**. Wild type was grown under normal (7H9) and salt stress conditions (150 mM NaCl, and 150 mM NH_4_Cl). Total RNA was extracted to synthesize cDNA. Relative expressions of *kdpF, kdpD*, and *kdpE* genes were studied by RT-qPCR using the 2^−ΔΔCt^ method. Data represent the averages of biological triplicates. Error bars indicate standard deviation. *sigA* was used as reference gene. Wild type strain grown under normal conditions (7H9 medium) was used as control to compare relative expressions.

Figure S10 **Expression of other K^**+**^ uptake loci in wild type and ***kdp*** mutants**. Genes of Trk and Trk related system such as *trkA* (*MSMEG_2771*), *trkB* (*MSMEG_2769*), TrkA domain-containing protein (*MSMEG_3665*), and ion channel membrane protein (*MSMEG_1945*) were studied in this experiment. Wild type, Δ*kdpD* and Δ*kdpE* were grown under normal and low K^+^ condition. Total RNA was extracted to synthesize cDNA. Relative expression was studied using the 2^−ΔΔCt^ method. Data represents the averages of biological triplicates. Error bars indicate standard deviation. *sigA* was used as a reference gene. Relative expression of above genes under K^+^ limiting conditions was determined in comparison with normal K^+^ conditions.

Table S1 **List of primers used in this study**.

Table S2 **Responses of Δ***kdpE*** under various stress conditions**.
