# Supplementary figures and images for: Regulation of Inducible Potassium Transporter KdpFABC by the KdpD/KdpE Two-Component System in Mycobacterium smegmatis
Source: Front Microbiol. 2017 Apr 24;8:570. doi: 10.3389/fmicb.2017.00570 (PMC5401905; doi:10.3389/fmicb.2017.00570)

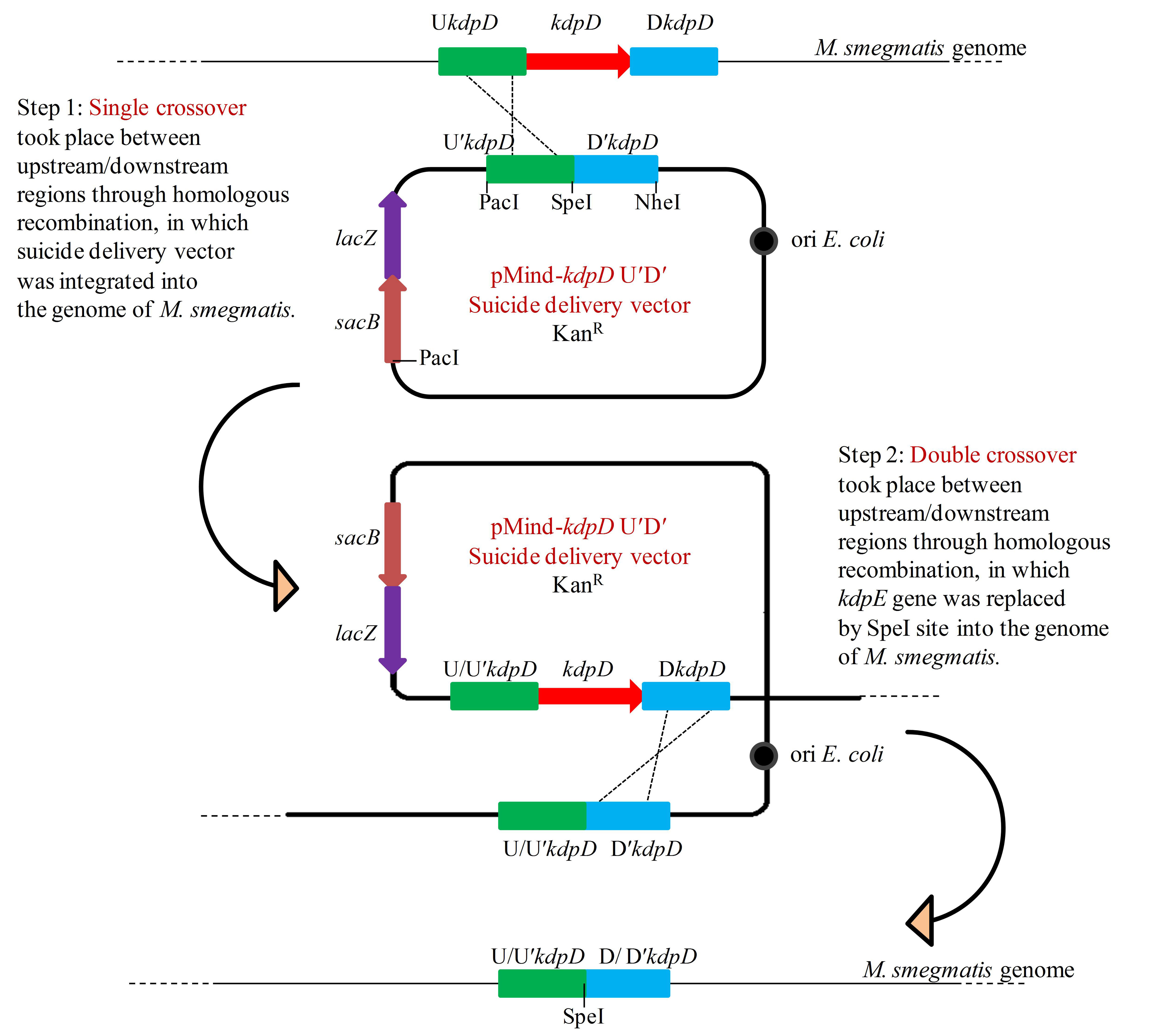

Supplement: Figure S1 — Schematic diagram of kdpD gene knock-out procedure in M. smegmatis MC2155. The kdpD mutation was carried out by the method of homologous recombination as described earlier (Yang et al., 2012). The 800-bp upstream and downstream regions flanking the target kdpD gene were amplified by PCR, purified and transferred into pMD19-T for sequence verification. These fragments were cloned into pMind vector (containing a hygromycin marker) at specific cloning sites. For ΔkdpD, hygromycin marker was excised from vector to create unmarked mutation. The resulting plasmid containing upstream and downstream regions was digested with PacI enzyme to insert the sacB-lacZ cassette from pGOAL17. This final suicide delivery vector was then electroporated into M. smegmatis MC2155 competent cells after 5 min direct exposure to UV radiation, and plated on 7H10 medium containing kanamycin (30 μg/mL) and X-gal (50 μg/mL) for single crossover (SCO). The obtained SCO colonies were allowed to grow in 7H9 broth without antibiotics to facilitate double crossover (DCO). One hundred microliters of culture was then plated on 7H10 medium containing 10% sucrose and X-gal (50 μg/mL). Resulting white colonies were tested for kanamycin susceptibility. [file Image1.JPEG]

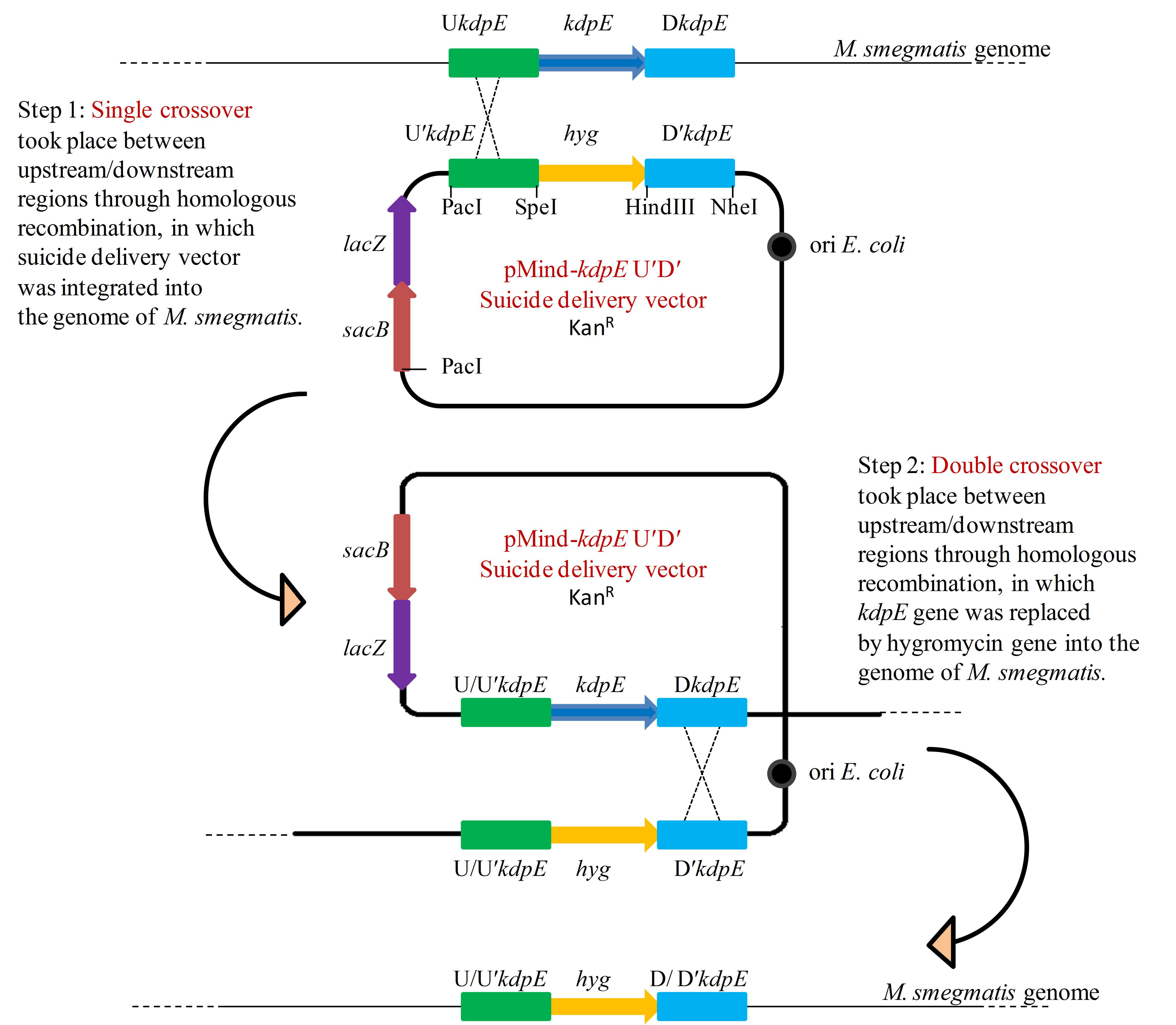

Supplement: Figure S2 — Schematic diagram of kdpE gene knock-out procedure in M. smegmatis MC2155. The kdpE knock-out was performed by the method of homologous recombination as described earlier (Yang et al., 2012). The 800-bp upstream and downstream regions flanking the target kdpE gene were amplified by PCR, purified and transferred into pMD19-T for sequence verification. These fragments were cloned into pMind vector (containing a hygromycin marker) at specific cloning sites. For kdpE gene, marked mutation method was used. The resulting plasmid containing upstream and downstream regions was digested with PacI enzyme to insert the sacB-lacZ cassette from pGOAL17. This final suicide delivery vector was then electroporated into M. smegmatis MC2155 competent cells after 5 min direct exposure to UV radiation and plated on 7H10 medium containing kanamycin (30 μg/mL), X-gal (50 μg/mL), and hygromycin B (100 μg/mL) for SCO. The obtained SCO colonies were allowed to grow in 7H9 broth without antibiotics to facilitate DCO and 100 μl culture was spread over 7H10 medium containing 10% sucrose, X-gal (50 μg/mL) and hygromycin B (100 μg/mL). Resulting white colonies were tested for kanamycin susceptibility. [file Image2.JPEG]

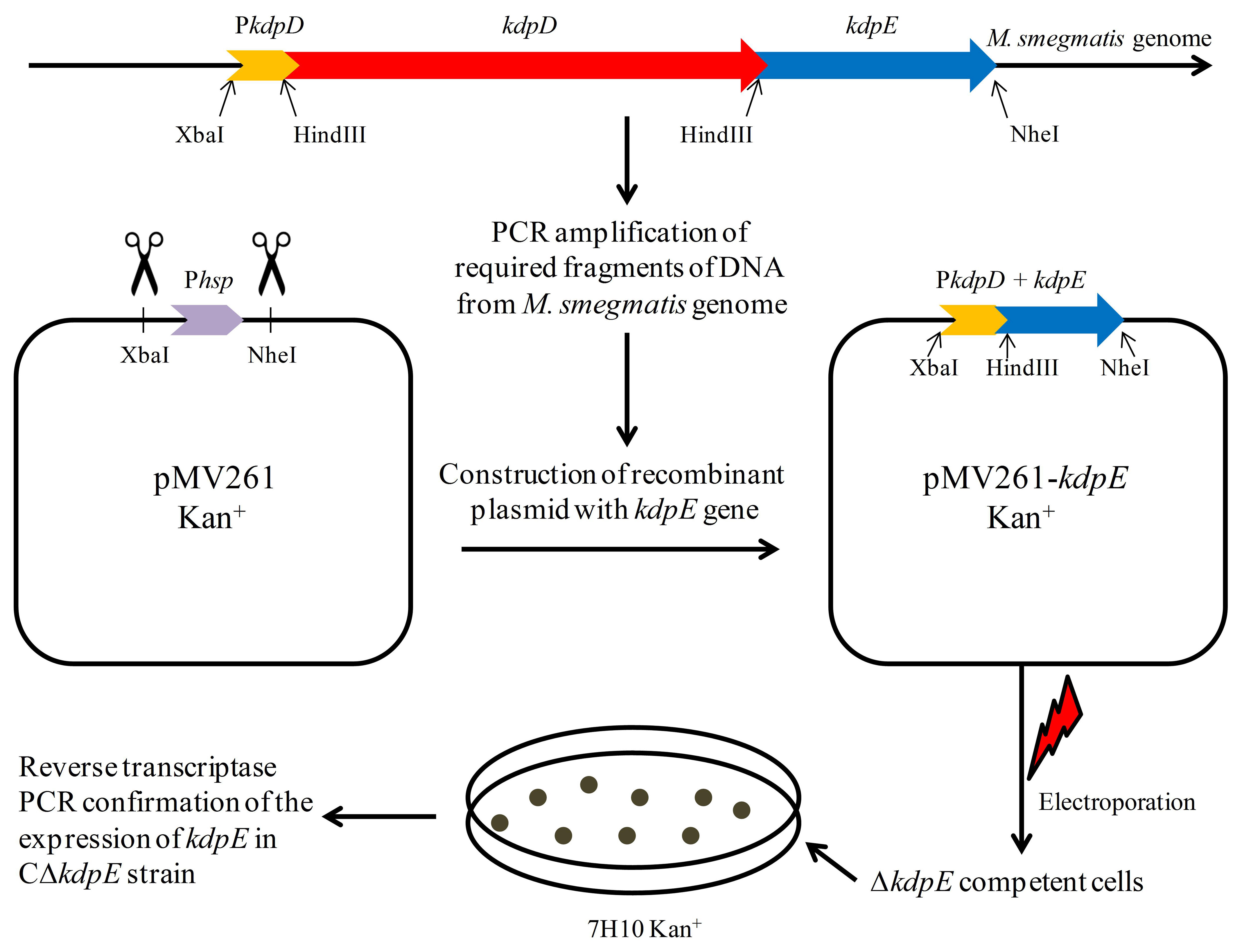

Supplement: Figure S3 — Construction of complementary strain of kdpE. Promoter region (PkdpD) was amplified by PCR using CP-D-F and CP-D-R primers (Table S1) and confirmed by sequencing. The hsp60 promoter (Phsp) in expression plasmid pMV261 (Yang et al., 2012) was replaced by PkdpD followed by insertion of kdpE gene that was amplified by primers C-kdpE-F and C-kdpE-R (Table S1). The resulting recombinant plasmid was confirmed by restriction endonucleases and PCR amplification. Plasmid was then electroporated into ΔkdpE competent cells. Resulting colonies were plated on 7H10 with kanamycin (30 μg/mL), and confirmed by RT-PCR for the expression of kdpE gene in CΔkdpE strain. [file Image3.JPEG]

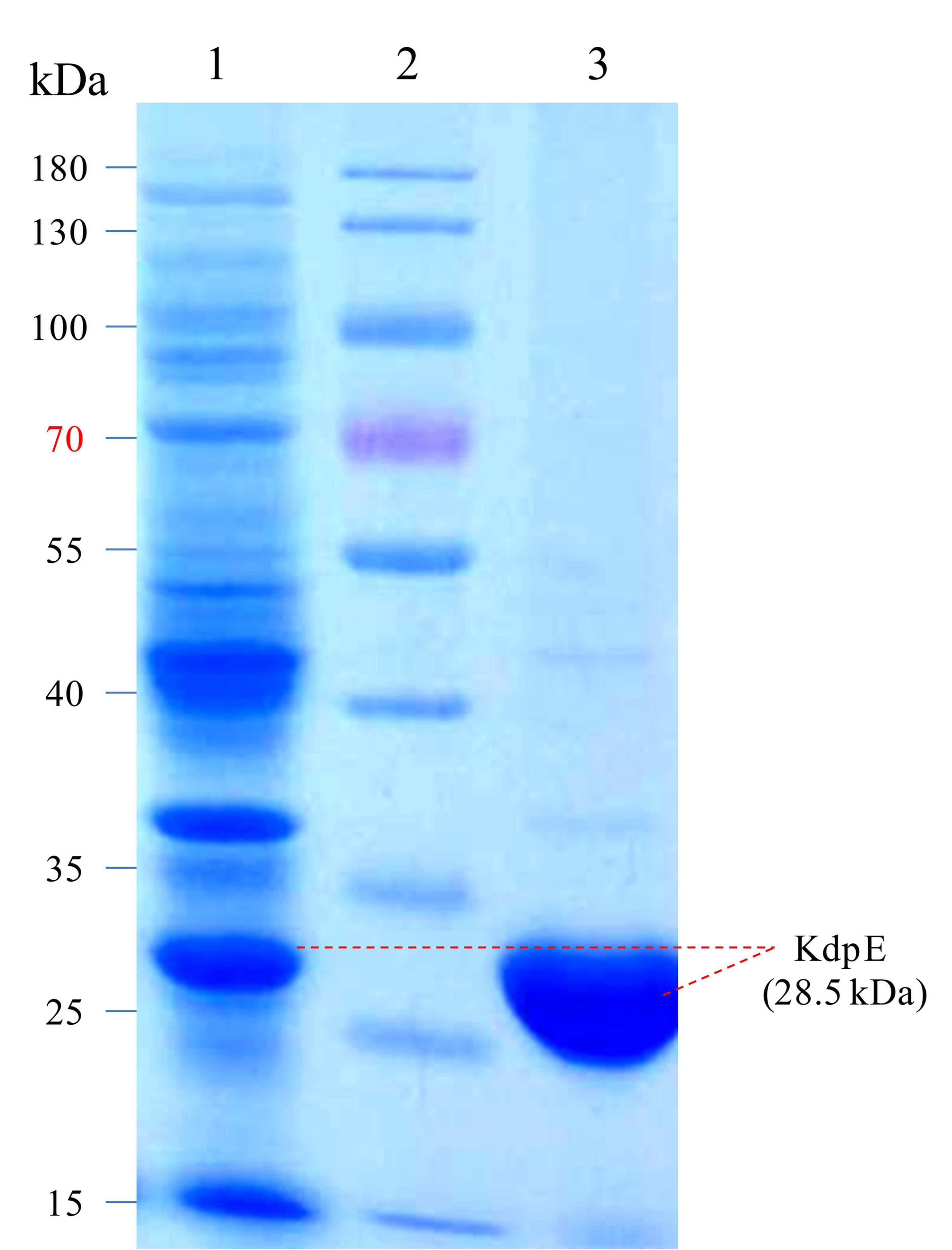

Supplement: Figure S4 — Heterologous expression of KdpE in E. coli BL21(DE3)-pET28-kdpE. KdpE protein from M. smegmatis genome was amplified and cloned into pET28a(+) to obtain expression plasmid pET28-kdpE that was transferred to E. coli BL21(DE3) to obtain the over-expression strain BL21(DE3)-pET28-kdpE. Isopropyl β-D-1-thiogalactopyranoside (IPTG) was added to the culture when OD600 reached to 0.5 and incubated at 28°C for 12 h for protein expression. Cells were harvested and re-suspended in lysis buffer (20 mM imidazole, 20 mM tris-base, 1 M NaCl) and lysed by ultrasonication. Crude cell lysate was centrifuged to remove debris and supernatant was used to purify 6 × His KdpE by using affinity column (Ni-NTA, GenScript). Cell lysate and purified KdpE protein were mixed with 2 × SDS loading buffer, boiled and centrifuged to run on 12% SDS PAGE. Lane 1: cell lysate, lane 2: protein marker, lane 3: purified KdpE protein (28.5 kDa). [file Image4.JPEG]

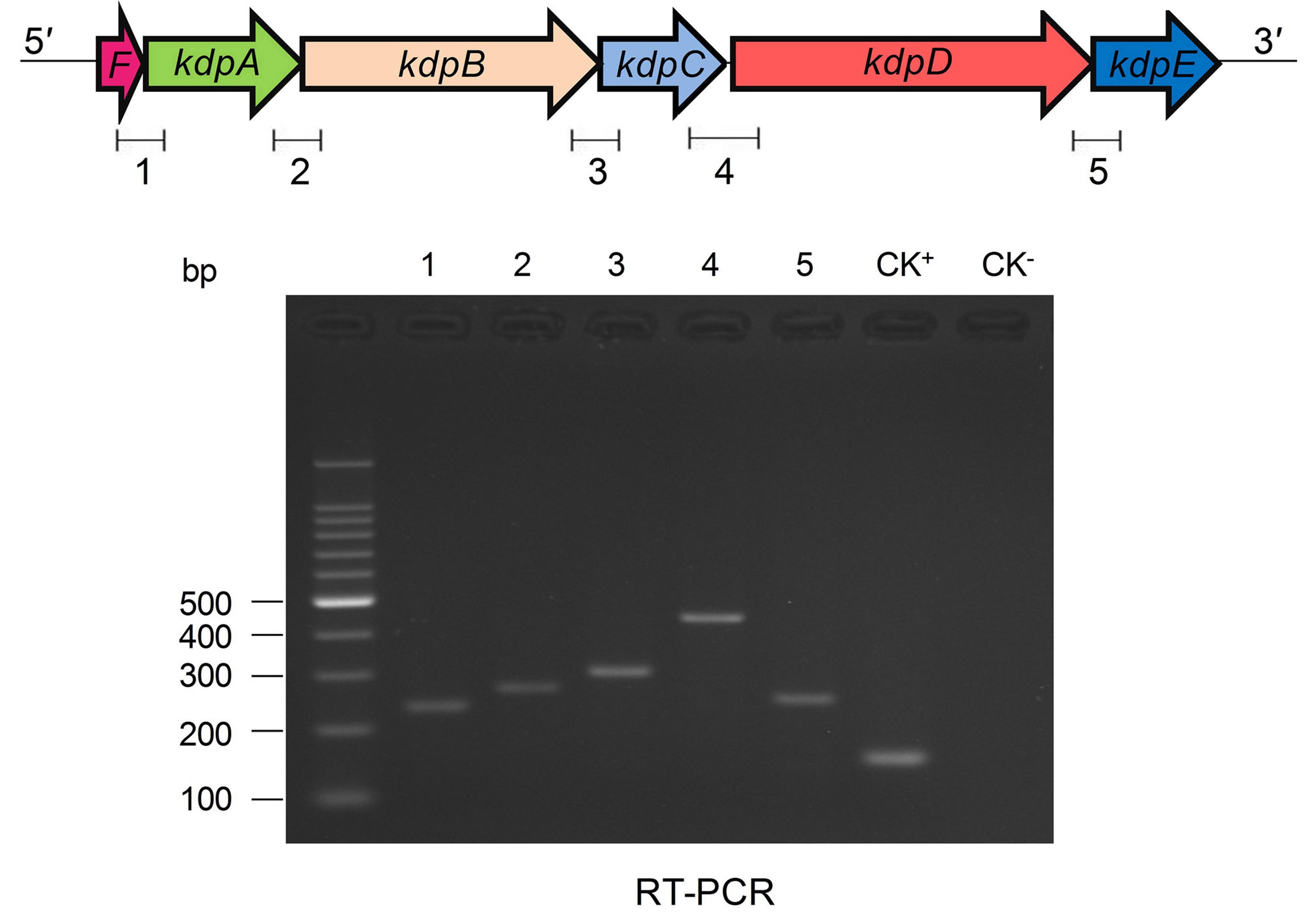

Supplement: Figure S5 — Co-transcription of kdpFABC and kdpDE operons under K+ limiting conditions. Genomic organization of the kdpFABC and kdpDE genes and small regions amplified in RT-PCR are shown by numbered short lines. Amplified PCR products obtained from K+ limiting cDNA were separated on 1% agarose gel. CK+ denotes amplification of a segment of sigA which was used as positive control, while CK− denotes PCR mixture without cDNA which was used as negative control. [file Image5.jpg]

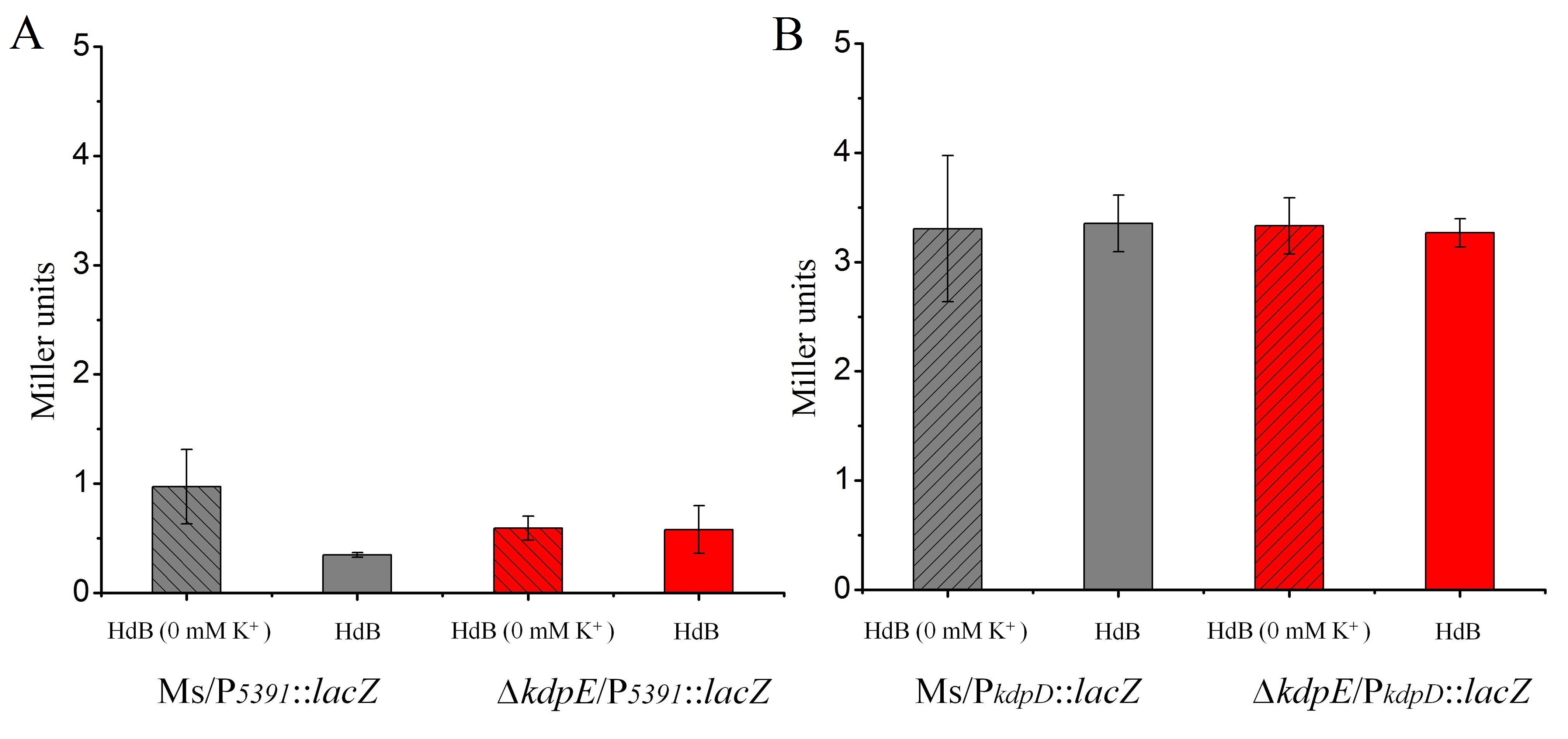

Supplement: Figure S6 — Promoter functionality β-galactosidase assays. (A) About 500 bp upstream region of MSMEG_5391 was taken as candidate promoter (P5391) and fused with promoter less lacZ gene to construct plasmid pMV261-P5391::lacZ. Newly constructed plasmid pMV261-P5391::lacZ was transformed into wild type and ΔkdpE. β-galactosidase activity was determined under normal and 0 mM K+ condition. (B) To study induction of promoter of kdpDE (PkdpD) under normal and 0 mM K+, about 500 bp upstream region of kdpDE operon was fused with promoter less lacZ and the newly constructed plasmid pMV261-PkdpD::lacZ was transformed into wild type and ΔkdpE. β-galactosidase activity was determined under normal and 0 mM K+ condition. Data represents the averages of biological triplicates. Error bars indicate standard deviation. [file Image6.JPEG]

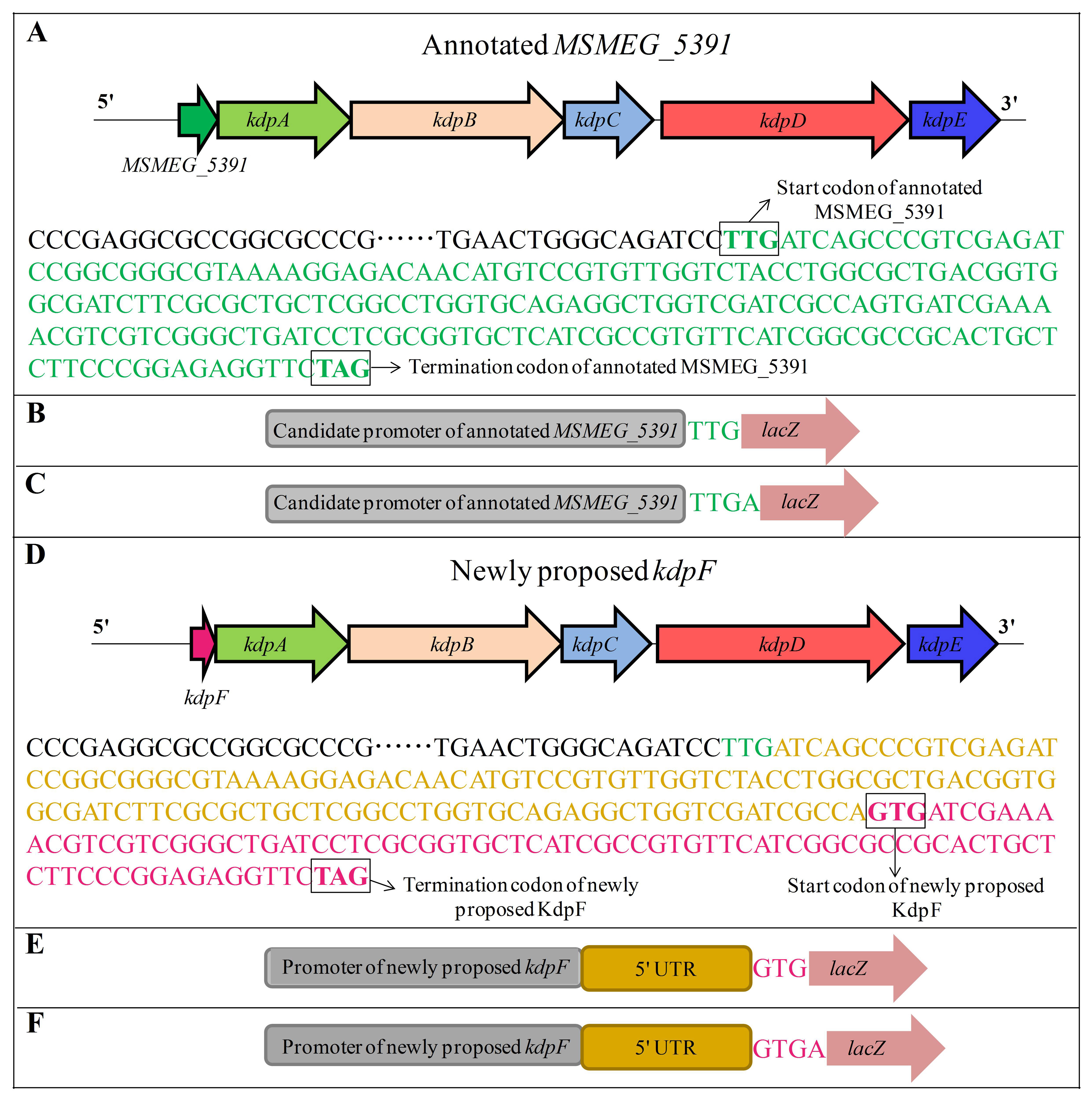

Supplement: Figure S7 — Design of promoter::lacZ vectors constructed for the identification of exact promoter sequence and kdpF identification. (A) The organization of annotated MSMEG_5391 with nucleotide sequence is shown in green. The start and termination codons of annotated MSMEG_5391 are boxed in black. (B) The 300 bp candidate promoter region upstream of annotated MSMEG_5391 gene with the start codon of MSMEG_5391 (TTG) fused with lacZ. (C) The 300 bp candidate promoter region upstream of MSMEG_5391 with the start codon of MSMEG_5391 plus one extra nucleotide (TTGA) to induce a frame-shifted lacZ. (D) The organization of newly proposed kdpF with nucleotide sequence is shown in magenta. The start and termination codons of newly proposed kdpF are boxed in black. (E) The PkdpF plus 5′-UTR of newly proposed kdpF with start codon (GTG) fused with lacZ. (F) The PkdpF plus 5′-UTR of newly proposed kdpF with start codon plus one extra nucleotide (GTGA) to induce frame shifted lacZ. [file Image7.JPEG]

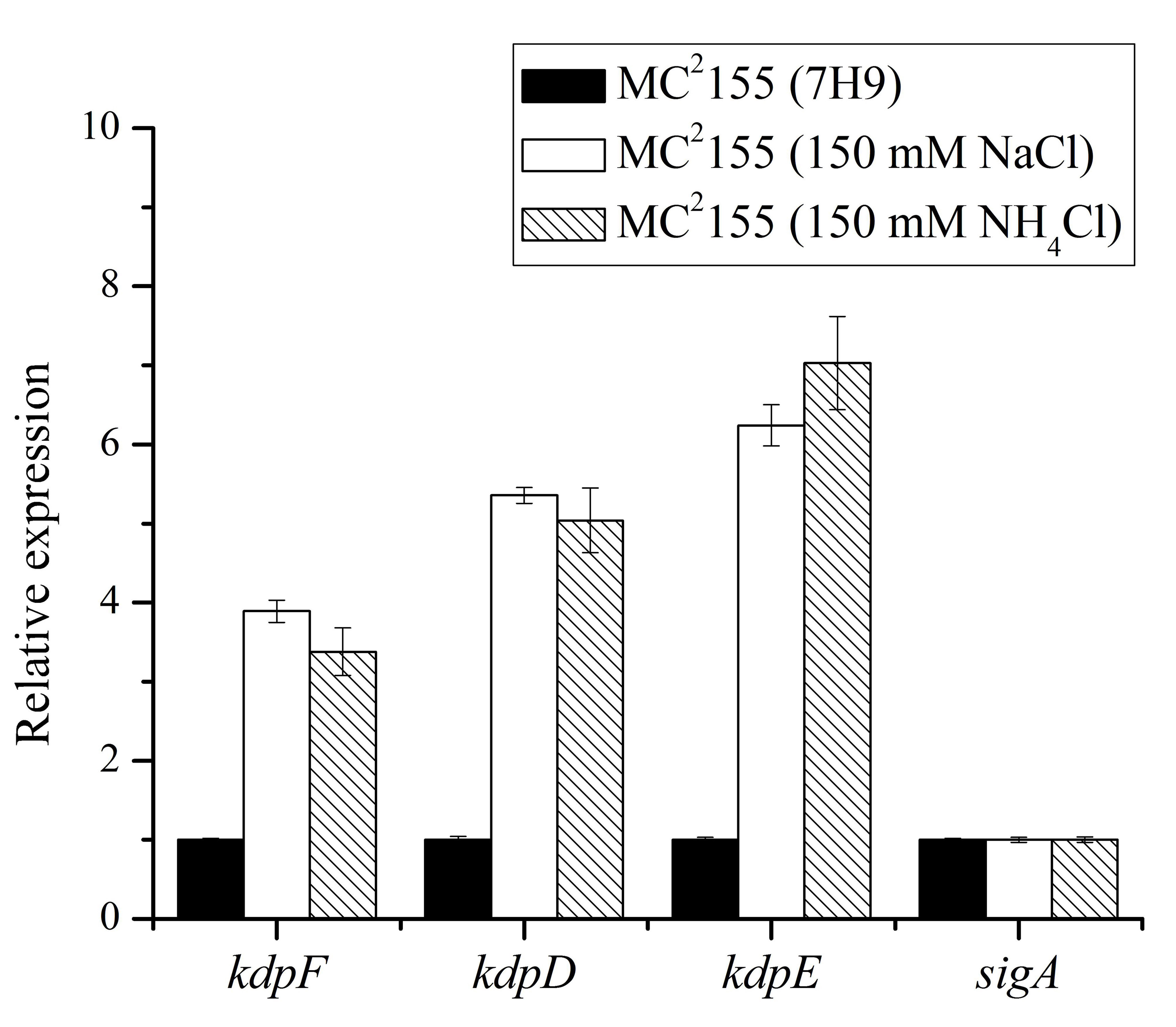

Supplement: Figure S9 — Induction of kdpF and kdpDE genes under osmotic stress. Wild type was grown under normal (7H9) and salt stress conditions (150 mM NaCl, and 150 mM NH4Cl). Total RNA was extracted to synthesize cDNA. Relative expressions of kdpF, kdpD, and kdpE genes were studied by RT-qPCR using the 2−ΔΔCt method. Data represent the averages of biological triplicates. Error bars indicate standard deviation. sigA was used as reference gene. Wild type strain grown under normal conditions (7H9 medium) was used as control to compare relative expressions. [file Image9.JPEG]

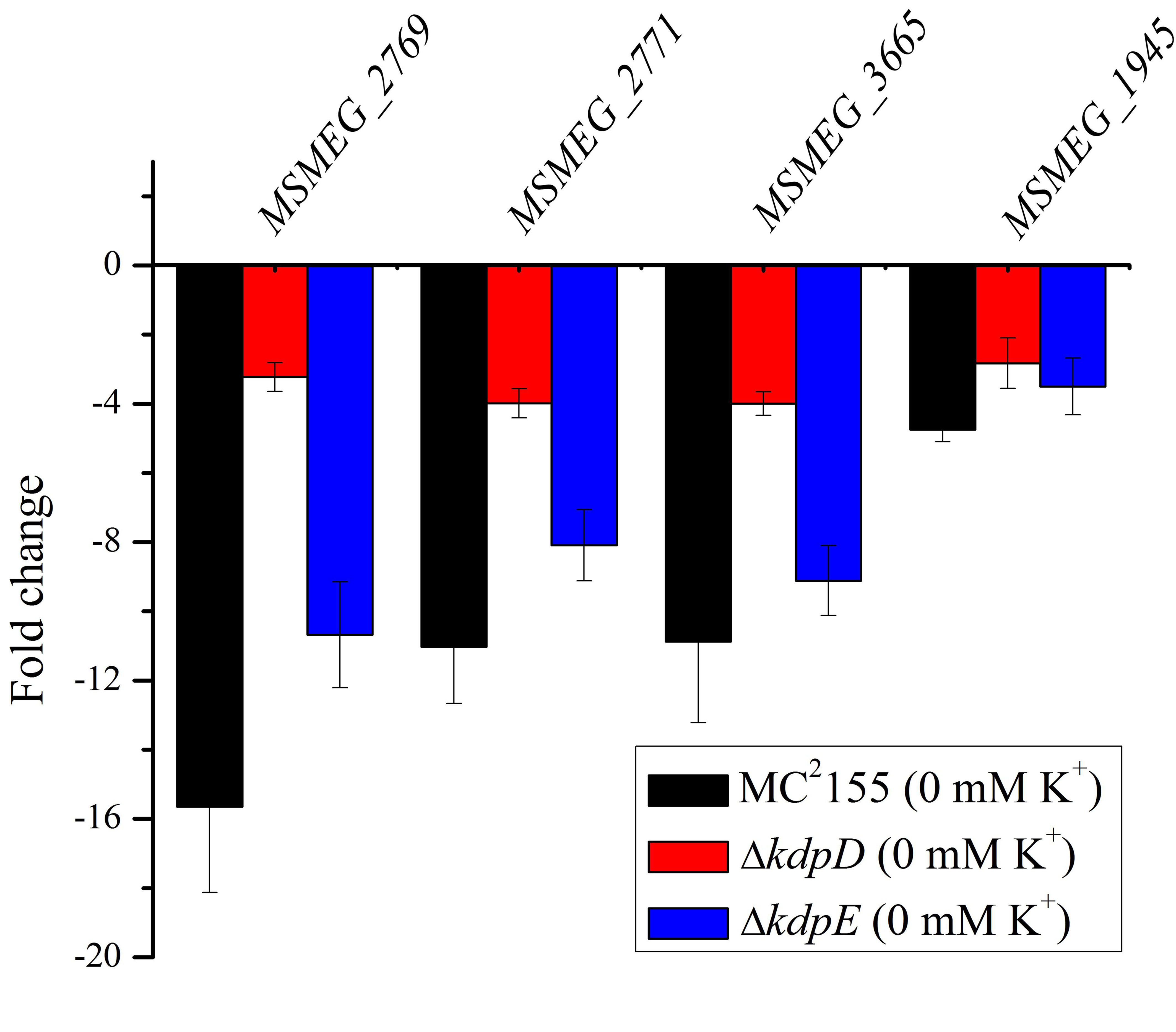

Supplement: Figure S10 — Expression of other K+ uptake loci in wild type and kdp mutants. Genes of Trk and Trk related system such as trkA (MSMEG_2771), trkB (MSMEG_2769), TrkA domain-containing protein (MSMEG_3665), and ion channel membrane protein (MSMEG_1945) were studied in this experiment. Wild type, ΔkdpD and ΔkdpE were grown under normal and low K+ condition. Total RNA was extracted to synthesize cDNA. Relative expression was studied using the 2−ΔΔCt method. Data represents the averages of biological triplicates. Error bars indicate standard deviation. sigA was used as a reference gene. Relative expression of above genes under K+ limiting conditions was determined in comparison with normal K+ conditions. [file Image10.JPEG]
