# Supplementary material for: Regulation of Inducible Potassium Transporter KdpFABC by the KdpD/KdpE Two-Component System in Mycobacterium smegmatis
Source: Front Microbiol. 2017 Apr 24;8:570. doi: 10.3389/fmicb.2017.00570 (PMC5401905; doi:10.3389/fmicb.2017.00570)
Supplement: Figure S8 — Organization of kdpFABC and kdpDE operons in different mycobacterial species. The positioning of kdpFABC and kdpDE operons in different mycobacterial species are shown in gene locus tags. Colored arrows showed different genes, with kdpF in magenta, kdpA in light green, kdpB in light orange, kdpC in light blue, kdpD in red, and kdpE in dark blue. Arrow head represents gene direction. Most of the kdpF sequences shown are identified in this study by sequence homology, and locus tags of kdpF are designated only to those species in which kdpF was previously and correctly annotated by NCBI/KEGG. In all species of mycobacteria that are analyzed in this study, the kdpDE operon is located adjacent to kdpFABC operon with the exception of M. chubuense, in which two operons are distantly located in same orientation. In case of M. chubuense, two slanted red lines indicate that two operons are not adjacent to each other. [file Image8.pdf]

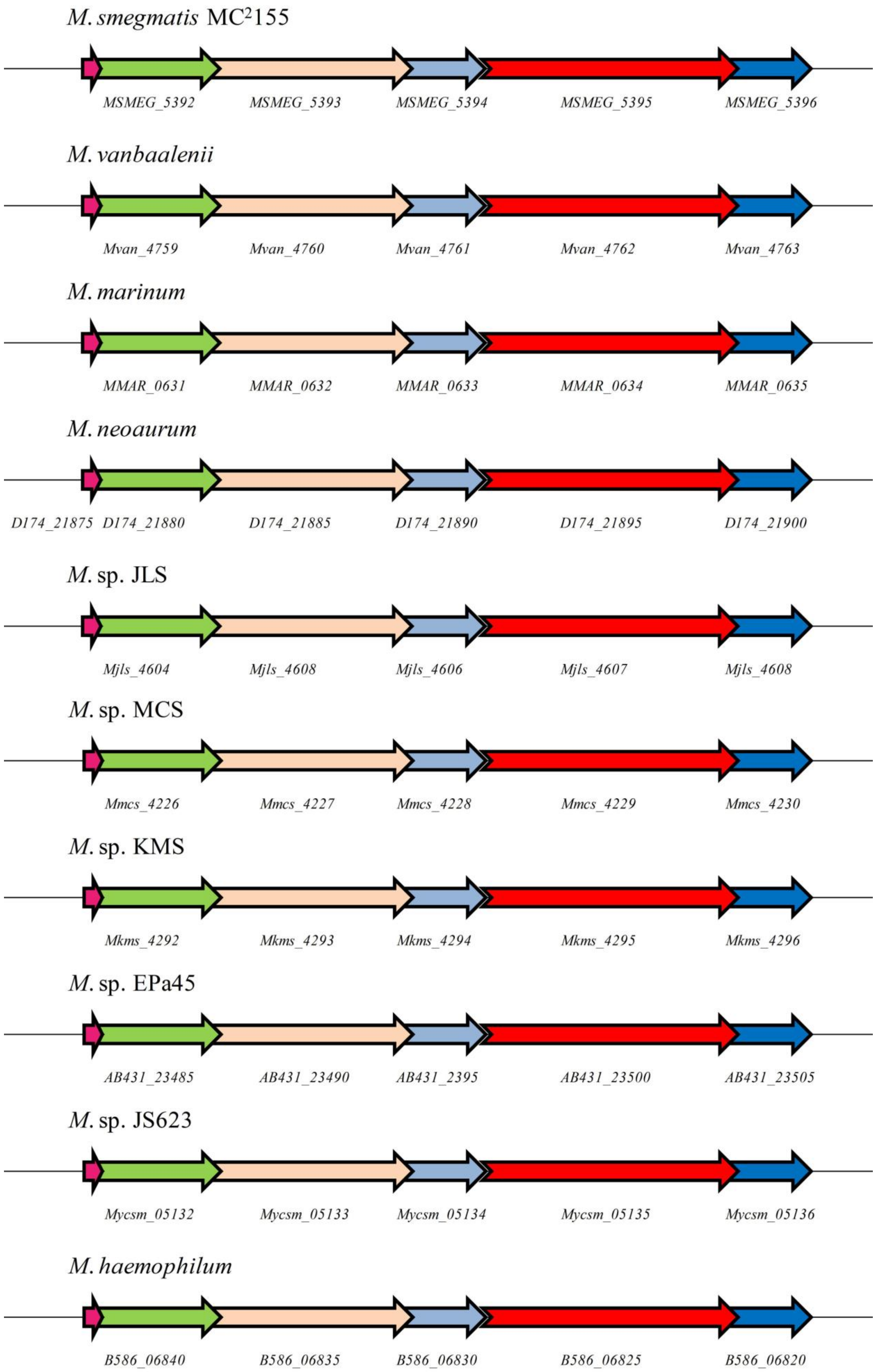
M. smegmatis MC^{2}155
MSMEG_5392
MSMEG_5393
MSMEG_5394
MSMEG_5395
MSMEG_5396
M. vanbaalenii
Mvan_4759
Mvan_4760
Mvan_4761
Mvan_4762
Mvan_4763
M. marinum
MMAR_0631
MMAR_0632
MMAR_0633
MMAR_0634
MMAR_0635
M. neoaurum
D174_21875
D174_21880
D174_21885
D174_21890
D174_21895
D174_21900
M. sp. JLS
Mjls_4604
Mjls_4608
Mjls_4606
Mjls_4607
Mjls_4608
M. sp. MCS
Mmcs_4226
Mmcs_4227
Mmcs_4228
Mmcs_4229
Mmcs_4230
M. sp. KMS
Mkms_4292
Mkms_4293
Mkms_4294
Mkms_4295
Mkms_4296
M. sp. EPa45
AB431_23485
AB431_23490
AB431_2395
AB431_23500
AB431_23505
M. sp. JS623
Mycsm_05132
Mycsm_05133
Mycsm_05134
Mycsm_05135
Mycsm_05136
M. haemophilum
B586_06840
B586_06835
B586_06830
B586_06825
B586_06820

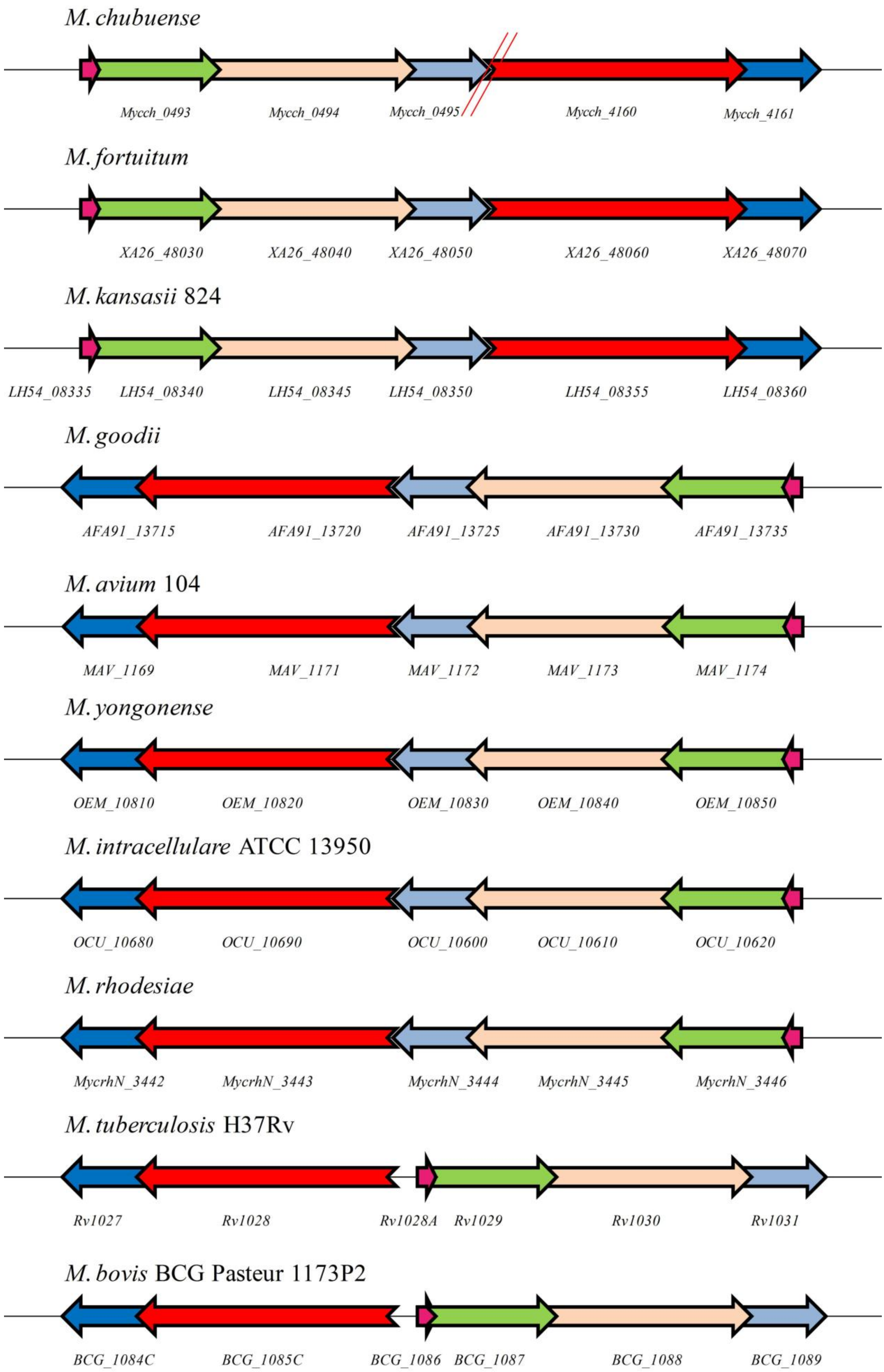
M. chubuense
Mycch_0493
Mycch_0494
Mycch_0495
Mycch_4160
Mycch_4161
M. fortuitum
XA26_48030
XA26_48040
XA26_48050
XA26_48060
XA26_48070
M. kansasii 824
LH54_08335
LH54_08340
LH54_08345
LH54_08350
LH54_08355
LH54_08360
M. goodii
AFA91_13715
AFA91_13720
AFA91_13725
AFA91_13730
AFA91_13735
M. avium 104
MAV_1169
MAV_1171
MAV_1172
MAV_1173
MAV_1174
M. yongonense
OEM_10810
OEM_10820
OEM_10830
OEM_10840
OEM_10850
M. intracellulare ATCC 13950
OCU_10680
OCU_10690
OCU_10600
OCU_10610
OCU_10620
M. rhodesiae
MycrhN_3442
MycrhN_3443
MycrhN_3444
MycrhN_3445
MycrhN_3446
M. tuberculosis H37Rv
Rv1027
Rv1028
Rv1028A
Rv1029
Rv1030
Rv1031
M. bovis BCG Pasteur 1173P2
BCG_1084C
BCG_1085C
BCG_1086
BCG_1087
BCG_1088
BCG_1089
