# Supplementary material for: Regulation of Inducible Potassium Transporter KdpFABC by the KdpD/KdpE Two-Component System in Mycobacterium smegmatis
Source: Front Microbiol. 2017 Apr 24;8:570. doi: 10.3389/fmicb.2017.00570 (PMC5401905; doi:10.3389/fmicb.2017.00570)
Supplement: Table S1 — List of primers used in this study. [file Table1.PDF]

Table S1. List of primers used in this study

Primers	Primer sequences (5'– 3')	Purposes
kdpE-up-F	CCTTAATTAAGAGTTGCCCGCACGCGACAGGCGTGTGTT	*kdpE* knockout
kdpE-up-R	GGACTAGTTCATGCCGGGACCGCCAGTTCGATCTC	*kdpE* knockout
kdpE-dw-F	CCCAAGCTTCGTCCCTCGCCGAGCAGACATAAAAC	*kdpE* knockout
kdpE-dw-R	GGCTAGCCGGCATGGGCATCGACAAGCCGAAT	*kdpE* knockout
kdpD-up-F	CCTTAATTAACCTCGGCTACCCGGTGTTCAT	*kdpD* mutation
kdpD-up-R	GGACTAGTATGACCATCATCCATGCGCG	*kdpD* mutation
kdpD-dw-F	CCCAAGCTTCTGCTCGCGGTGGCAGTC	*kdpD* mutation
kdpD-dw-R	GGCTAGCAGGCCGCCGTCGGCCAGT	*kdpD* mutation
PU-F	GGAGGCCGGAGACGAGTTTT	Δ*kdpE* confirmation
PD-R	ACGACGACGAGGTTGACGTG	Δ*kdpE* confirmation
HYG-DW-F	CCTGGTGCAACTGCATCTCAA	Δ*kdpE* confirmation
HYG-UP-R	GCGTAGGAATCATCCGAATCAATA	Δ*kdpE* confirmation
KdpE-DE3-F	CGCGGATCCACCGCCCCGATCAAGACCC	KdpE protein expression
KdpE-DE3-R	CCCAAGCTTTCACGCCTCGAAGCGGTAACCCAT	KdpE protein expression
P-BG-F	GCTCTAGAGCAACCGCCTTGTCGCGCA	β-galactosidase assay
P-TTG-R	CCCAAGCTTTCAAGGATCTGCCCAGTT	β-galactosidase assay
P-TTGA-R	CCCAAGCTTATCAAGGATCTGCCCAGTT	β-galactosidase assay
P-GTG-R	CCCAAGCTTTCACTGGCGATCGACCAG	β-galactosidase assay
P-GTGA-R	CCCAAGCTTATCACTGGCGATCGACCAG	β-galactosidase assay
P-FABC-F	GCTCTAGACAGACCGACCGAGCAGAAC	Promoter activity assay
P-FABC-R	CCCAAGCTTGGATCTGCCCAGTTCAGG	Promoter activity assay
P-KdpD-F	GCTCTAGAGGGACGTCGACGGGATCAGC	Promoter activity assay
P-KdpD-R	CCCAAGCTTATGACCATCATCCATGCGC	Promoter activity assay
CP-D-F	GCTCTAGAGCGGTGCGCGGCCGTTCTG	Δ*kdpE* complementation
CP-D-R	CCCAAGCTTCGACGCCCGCGTAGTCAC	Δ*kdpE* complementation
C-kdpE-F	CCCAAGCTTACCGCCCCGATCAAGACCC	Δ*kdpE* complementation
C-kdpE-R	GGCTAGCTCAATGGTGATGGTGATGGTGCGCCTCGAAG CGGTAACC	Δ*kdpE* complementation
P5391-FAM-F	CCCGAGGCGCCGGCGCCCGG	EMSA
P5391-300-R	GGATCTGCCCAGTTCAGGACCGCG	EMSA
P5395-300-F	GGCCCCGTTCCTCTACACGT	EMSA
P5395-300-R	ATGACCATCATCCATGCGCGG	EMSA
FPC-33-F	ACCGTAAAGAAACCATCAAGGCCGCGTTCGCGG	Footprint confirming EMSA
FPC-33-R	CCGCGAACGCGGCCTTGATGGTTTCTTTACGGT	Footprint confirming EMSA
FPC-28-F	CCGTAAAGAAACCATCAAGGCCGCGTTC	Footprint confirming EMSA
FPC-28-R	GAACGCGGCCTTGATGGTTTCTTTACGG	Footprint confirming EMSA
FPC-26-F	CGTAAAGAAACCATCAAGGCCGCGTT	Footprint confirming EMSA
FPC-26-R	AACGCGGCCTTGATGGTTTCTTTACG	Footprint confirming EMSA
FPC-25-F	CCGTAAAGAAACCATCAAGGCCGCG	Footprint confirming EMSA

FPC-25-R	CGCGGCCTTGATGGTTTCTTTACGG	Footprint confirming EMSA
FPC-24-F	GTAAAGAAACCATCAAGGCCGCGT	Footprint confirming EMSA
FPC-24-R	ACGCGGCCTTGATGGTTTCTTTAC	Footprint confirming EMSA
FPC-23-F	CCGTAAAGAAACCATCAAGGCCG	Footprint confirming EMSA
FPC-23-R	CGGCCTTGATGGTTTCTTTACGG	Footprint confirming EMSA
FPC-22-F	CCGTAAAGAAACCATCAAGGCC	Footprint confirming EMSA
FPC-22-R	GGCCTTGATGGTTTCTTTACGG	Footprint confirming EMSA
FPC-20-F	CCGTAAAGAAACCATCAAGG	Footprint confirming EMSA
FPC-20-R	CCTTGATGGTTTCTTTACGG	Footprint confirming EMSA
FPC-18-F	AAGAAACCATCAAGGCCG	Footprint confirming EMSA
FPC-18-R	CGGCCTTGATGGTTTCTT	Footprint confirming EMSA
FPC-17-F	CCGTAAAGAAACCATCA	Footprint confirming EMSA
FPC-17-R	TGATGGTTTCTTTACGG	Footprint confirming EMSA
KdpF-F	CGGGCGTAAAAGGAGACAAC	RT-q PCR assay
KdpF-R	ACGTTTTCGATCACTGGCGA	RT-q PCR assay
KdpA-F	GCGCCGAACGTTTGATCTAC	RT-q PCR assay
KdpA-R	GTTGGTGTTGGTGACGAAGC	RT-q PCR assay
KdpB-F	CAAGGCATGAAGCAGCGTTT	RT-q PCR assay
KdpB-R	TCAATGAGCTTGGTCGGGTC	RT-q PCR assay
KdpC-F	GAAGGTGTACGGGTCGAGTG	RT-q PCR assay
KdpC-R	GCGATGTTGAGTTCCAGCAC	RT-q PCR assay
KdpD-F	ATGTCCACATGGTGACCCAC	RT-q PCR assay
KdpD-R	AGGAACGGATCGAGAATCGC	RT-q PCR assay
KdpE-F	TCGAGACCACATCGTTCACC	RT-q PCR assay
KdpE-R	GGTCGTCTTCGAGCTTACGG	RT-q PCR assay
SigA-F	CGAGGAAGAAGAAGCTGATG	RT and RT-q PCR assay
SigA-R	CGTCTTTGCGTGCCTGTC	RT and RT-q PCR assay
KdpA-Inn-F	GTGATCGAAAACGTCGTCGG	RT-PCR assay
KdpA-Inn-R	ACCGATCACCCGGTAGATCA	RT-PCR assay
KdpB-Inn-F	GCGCGGGTTCAGCTTGCG	RT-PCR assay
KdpB-Inn-R	GCTCGGTCGGCACCCTGC	RT-PCR assay
KdpC-Inn-F	CCCTCAAGGGTGTCCGCTAC	RT-PCR assay
KdpC-Inn-R	GATGATCGATCCGTCGGCCT	RT-PCR assay
KdpD-Inn-F	CGTGGACCCGCAACGGGTA	RT-PCR assay
KdpD-Inn-R	GACAGCGGCCACCAGGTC	RT-PCR assay
KdpE-Inn-F	TGGGCCTGGGACTCTCGGTG	RT-PCR assay
KdpE-Inn-R	CTGTGCAACGCCTCGGCGC	RT-PCR assay
5’-RACEOuterF	CATGGCTACATGCTGACAGCCTA	5'-RACE analysis
5’-RACEOuterR	GGTGTAGACGCGGTACATGTAGT	5'-RACE analysis
5’-RACEInnerF	CGCGGATCCACAGCCTACTGATGATCAGTCGATG	5'-RACE analysis
5’-RACEInnerR	GGTGGGGGACACTAGAACCTCTCCGGG	5'-RACE analysis
