# Supplementary material for: Regulation of Inducible Potassium Transporter KdpFABC by the KdpD/KdpE Two-Component System in Mycobacterium smegmatis
Source: Front Microbiol. 2017 Apr 24;8:570. doi: 10.3389/fmicb.2017.00570 (PMC5401905; doi:10.3389/fmicb.2017.00570)
Supplement: Table S2 — Responses of ΔkdpE under various stress conditions. [file Table2.PDF]

Table S2. Responses of Δ*kdpE* under various stress conditions

Stresses condition	Time (h)	10^{-1}	10^{-2}	10^{-3}	10^{-4}	10^{-5}
Heat (50 °C)	0	+++	+++	++	++	+
	5	+++	+++	+++	+++	++
Cold (28 °C)	0	+++	+++	+++	+++	++
	8	+++	+++	+++	+++	++
Acidic pH 4.0	0	+++	+++	++	++	
	5	+++	+++	++	+	
Alkaline pH 9.0	0	+++	+++	+++	+++	++
	5	+++	+++	+++	+++	++
NaCl (5 mM)	0	+++	+++	+++	+++	++
	5	+++	+++	+++	+++	++
ddH_2O	0	+++	+++	+++	+++	++
	5	+++	+++	+++	++	+
H_2O_2 (2-5 mM)	0	+++	+++	+++	++	+
	2	+++	+++	+++	++	+
Ethanol (5 %)	0	+++	+++	+++	+++	++
	5	+++	+++	+++	+++	++
$NaNO_2$ (5 mM, pH 7.0)	0	+++	+++	+++	+++	++
	4	+++	+++	+++	+++	++
$NaNO_2$ (5 mM, pH 5.5)	0	+++	+++	+++	+++	++
	4	+++	+++	+++	+++	++

+++ = robust growth, ++ = compromised growth, + = attenuated growth

Bacterial strains were exposed to stress condition for different time intervals and then plated out on 7H10 media. There was no significant difference observed in the growth of wild type and mutant strains at most of the stresses studied. However, on the heat shock of 50 °C for 5 h, Δ*kdpE* was slightly resistant (red color). On contrary, under hypo-osmotic condition in H_2O and acidic pH 4.0 in citrate phosphate buffer for 5 h, Δ*kdpE* appeared to be slightly sensitive (green color).
